# Mitochondria-targeted aggregation induced emission theranostics: crucial importance of *in situ* activation†
†Electronic supplementary information (ESI) available. See DOI: 10.1039/c6sc02236g


**DOI:** 10.1039/c6sc02236g

**Published:** 2016-06-07

**Authors:** Weon Sup Shin, Min-Goo Lee, Peter Verwilst, Joung Hae Lee, Sung-Gil Chi, Jong Seung Kim

**Affiliations:** a Department of Chemistry , Korea University , Seoul , 136-701 , Korea . Email: jongskim@korea.ac.kr; b School of Life Sciences and Biotechnology , Korea University , Seoul , 136-701 , Korea . Email: chi6302@korea.ac.kr; c Korea Research Institute of Standards and Science , Daejeon 305-600 , Korea

## Abstract

Tissue selective targeting and specific suborganellular localization combined with an efficient pathology associated enzymatic activation of drugs in drug delivery systems may exhibit a clear advantage over conventional cancer treatment. Here, a mitochondria targeted aggregation induced emission (AIE) fluorophore further conjugated with an NAD(P)H:quinone oxidoreductase-1 (NQO1) cleavable masking unit showed preferential uptake in cancer cells and was selectively activated, resulting in bright AIE fluorescence and apoptosis *via* the caspase pathway, triggered by mitochondrial dysfunction. *In vivo* experimental data further support the conclusions from *in vitro* experiments, clearly showing the dependence of the therapy's success on both the suborganelle localization and specific *in situ* activation. And the site specific and enzyme dependent activation and aggregation was further supported by *in vivo* and *ex vivo* imaging. As a whole, the data comprised in this work represent a strong argument for the further development of this type of novel anticancer drugs.

## Abstract 2

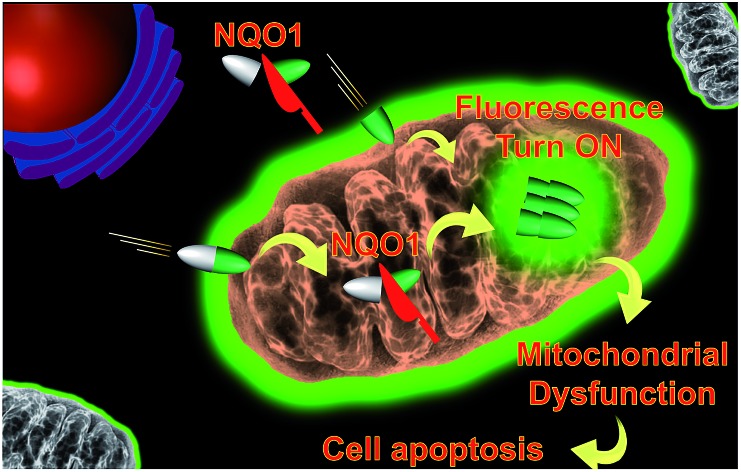
A mitochondria targeted AIE fluorophore was further decorated with an NQO1 cleavable masking unit and showed selective targeting to and activation in cancer cells resulting in bright AIE fluorescence and apoptosis triggered by mitochondrial dysfunction.

## Introduction

Despite astounding advances in the field of cancer treatment over the last decades, cancer therapeutics, often exhibiting suboptimal narrow therapeutic indexes, are inherently associated with severe side effects due to a lack of specificity for cancerous tissues and treatment often results in the development of drug resistance, as cancer cells are likely to be repeatedly exposed to non-lethal concentrations of drugs during the course of cancer treatment.^1^

In a quest to develop more specific cancer drugs, thus diminishing side effects and widening the therapeutic window of the drugs, targeted drug delivery has emerged as an efficient way to deliver drugs, utilizing specific cellular receptors, overexpressed on certain tumor tissues and other cancer-associated physiological differences.^2^ In recent years the specific targeting of not just the cancerous tissues, but the precise delivery of a cytotoxic agent at the subcellular level has been shown to hold great promise to further decrease side effects and combat multi-drug resistance.^3^

Mitochondria, widely known as the cell's powerhouse, are vital subcellular organelles in both normal and malignant cells. Upon the cells evolution into a pathological state, the mitochondria exhibit intricately interwoven signal transduction pathways, crucial to the survival and metastatic potential of cancer cells.^4^ Therefore, interrupting the metabolism of this organelle represents an elegant and efficient target for novel cancer therapies. Mitochondria exhibit significant cancer-associated dysfunctions, amongst them, the reduced activity of ATP synthase results in an increased membrane potential and the increased production of reactive oxygen species (ROS),^4^ representing yet another mechanism by which cancer cells may acquire drug resistance following redox adaptation.^5^

The transmembrane potential of mitochondria, with a negatively charged interior, attracts delocalized lipophilic cations, with the triphenylphosphonium group as the prototypical example, resulting in their electrophoretic transmembrane migration and upconcentration with reported concentration enhancements up to 500-fold.^6^ As cancerous cells contain mitochondria with increased mitochondrial potential and thus significantly higher mitochondrial upconcentration of delocalized lipophilic cations, the conjugation of these cations to cytotoxic agents opens the path towards increasing tumor specificities of anticancer agents, and thus the mitochondria represent a sensitive suborganellar targeting site with a convenient targeting mechanism.^3^,^6^

Key to developing more specific anticancer agents is not only exerting control over the tissue and organelle specificity of anticancer drugs, but also the presence of a mechanism to verify the delivery of the novel drugs, and whereas several strategies exist, the use of fluorescence is particularly interesting as a safe, non-invasive method, generally requiring relatively inexpensive experimental setups. Conventional approaches to this combination of therapy and diagnosis, known as small molecule based theranostic cancer treatment,^7^ entail the covalent linking of a fluorophore or profluorophore to the drug. A more atom-efficient way to achieve a theranostic platform, is using a drug, that upon delivery becomes fluorescent itself. Aggregation induced emission (AIE) fluorophores, a particular class of fluorophores, exhibiting increased fluorescence upon aggregation, as opposed to most fluorophores associated with quenched fluorescence upon aggregation, due to restricted intermolecular rotations in the self-assembled state,^8^ are ideally suited for this purpose. Furthermore, the specific aggregation of this type of fluorophore at mitochondrial sites has recently been shown to result in cell death due to disrupted mitochondrial metabolism, whilst signaling the event by fluorescence originating from those mitochondria.^9^

A second strategy to increase the specificity of anticancer drugs towards malignant tissues is the use of enzyme activatable prodrugs. Making use of the differential expression of various enzymes in cancerous cells and microenvironments, these masked cytotoxic drugs are predominately activated in the target tissues, thus lowering the potential of side-effects resulting from non-specific drug uptake and thus potentially widening the therapeutic window.^10^

NAD(P)H:quinone oxidoreductase-1 (NQO1) is an enzyme expressed throughout the cell and its expression is upregulated as a response to stress. The enzyme acts as a two-electron reductase towards various toxins such as quinone xenobiotics and the superoxide anion with concomitant consumption of either NADH or NADPH.^11^ In view of its stress induced upregulation it is not surprising that the enzyme is overexpressed in various cancerous tissues^11^ with ratios up to 50 fold in the case of human liver carcinoma.11*a* Furthermore, it is theorized that NQO1 upregulation may be an early event in the pathological transformation of normal cells to cancerous cells.^11^

**Scheme 1:**
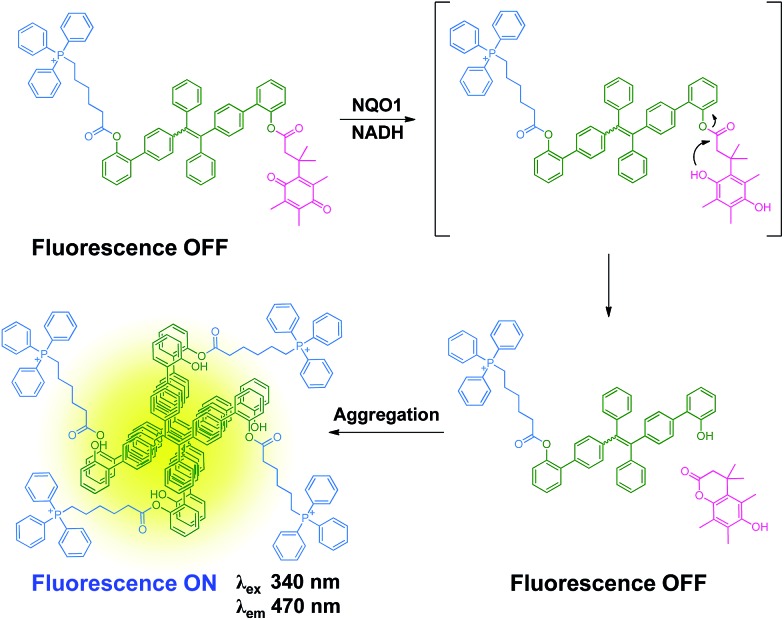
Proposed mechanism of the reaction of **1** with NQO1.

The combination of both tissue and/or organelle selective delivery and tissue specific enzyme gated activation of prodrugs dramatically increases the selectivity of a drug conjugate and in the current work we utilize this strategy on an AIE scaffold. Here, an AIE fluorophore is decorated with both a tumor and organelle targeting triphenylphosphonium moiety and a quinone based trigger,^12^ allowing the selective activation of the AIE self-assembly by the NQO1 enzyme. The proposed mechanism is depicted in Scheme 1 where the non-fluorescent prodrug **1** is targeted towards and upconcentrated in the mitochondria of cancer cells. The aggregates of **1** undergo rapid reduction of the quinone scaffold and subsequent cyclisation releases the active form of the AIE dye, signalled by fluorescence due to the elimination of a photo-induced electron transfer process between the AIE scaffold and the quinone trigger. The AIE aggregates, specifically localised to the mitochondria, subsequently induce the disruption of major metabolic pathways, finally culminating in the selective apoptosis of the targeted cells.

## Results and discussion

### Synthesis

**Scheme 2:**
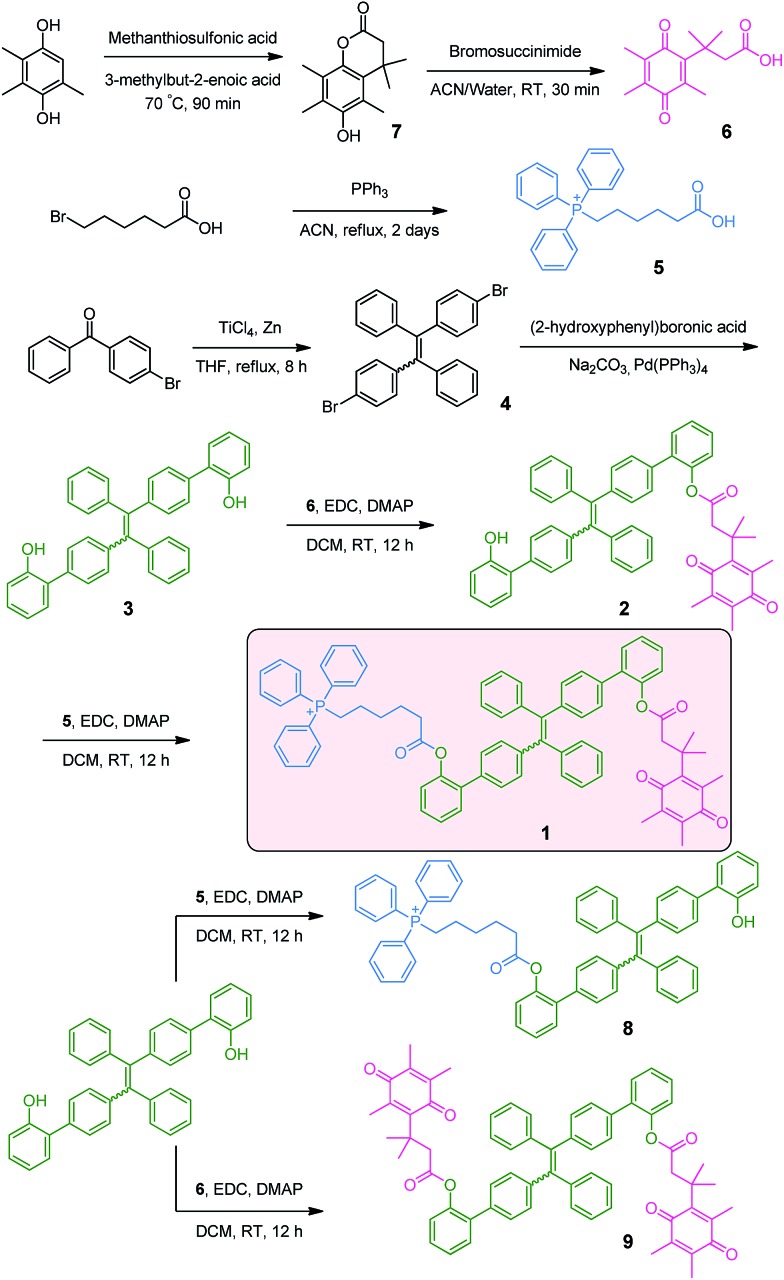
Synthetic route towards **1**.

As depicted in Scheme 2, the NQO1 trigger **6** was synthesized in two steps according to a previously reported procedure.^13^ The triphenylphosphonium-containing targeting moiety **5** was also obtained efficiently as previously described.12*g* The dibrominated tetraphenylethene precursor **4** was prepared *via* the McMurry coupling of 4-bromoacetophenone, resulting in a *cis*/*trans* ratio of 44/56 (data not shown), as previously reported.^14^ The *cis*/*trans* mixture was subsequently used as the scaffold for a Suzuki coupling reaction with (2-hydroxyphenyl)boronic acid, yielding the π-extended tetraphenylethene **3**. Finally, the mitochondria targeted NQO1 activatable AIE fluorophore **1**, as well as reference compounds **8** and **9**, lacking the NQO1 triggering unit and the mitochondria targeting unit, respectively, were available through the esterification of **3** using a carbodiimide coupling reagent. The identities of all compounds **1–6** were confirmed by ^1^H NMR, ^13^C NMR and ESI-MS data as provided in the ESI (Fig. S1–S10†). The purity of compounds **1**, **8** and **9** was further determined by HPLC to be 92.4%, 85.8% and 95.0%, respectively (Fig. S11†).

### DFT calculations

The relative energies of the frontier orbitals of both *cis* and *trans* isomers of the AIE core (Fig. S12a†) have been calculated by DFT calculations using the B3LYP hybrid functional at the 6-31G(d) level of theory in the gas phase. The calculations revealed relatively small differences in energy between them (Fig. S12b†), with energies for the HOMO being –5.31 and –5.37 eV, for the *cis* and *trans* isomer respectively and for the LUMO being –1.43 and –1.44 eV, for the *cis* and *trans* isomer respectively. This can be rationalised by a relatively small contribution of the *ortho*-substituted phenylacetate pendent arms, as can be seen from the electron distributions of the frontier molecular orbitals (Fig. S12c†).

### Solution studies

The absorption and emission spectra of **1**, **8** and **9** were recorded in methanol with increasing amounts of water (Fig. S13†). Whereas the absorption was largely independent of the solvent composition, a large solvent dependent fluorescence increase was observed for **8** only, resulting from efficient aggregation induced fluorescence at higher water concentrations. It is interesting to note that compounds **1** and **9**, both bearing the NQO1 trigger did not exhibit significant increases in fluorescence at higher concentrations of water, despite having a similar polarity as **8**. Taking the DFT calculation results of the quinone trigger into account (Fig. S12†), this observation is supported by the relative energies of the frontier orbitals of both the trigger and the AIE core (both *cis* and *trans* isomers), where the NQO1 trigger quenches the fluorescence of the AIE unit by an oxidative electron transfer (OeT)-type photoinduced electron transfer (PeT) effect, as previously reported for a naphthalimide-based NQO1 specific fluorophore.12*f*

**Fig. 1:**
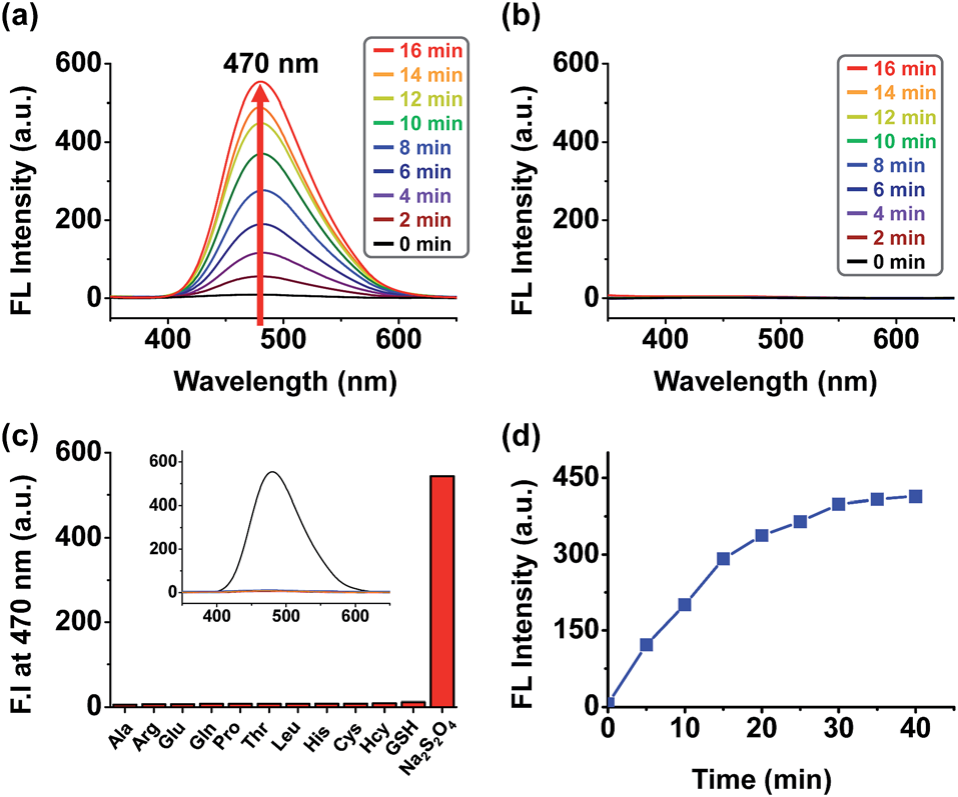
Fluorescence spectrum of a 20 μM solution of **1** (a) in PBS (pH 7.4) during a 15 min period upon reduction by 40 μM sodium dithionite (b) in MeOH during a 15 min period upon reduction by 40 μM sodium dithionite (*λ*_ex_ = 340 nm, slit = 5/5 *T* = 25 °C). (c) Fluorescence response assays for compound **1** (20 μM) and various biological reductants, including thiol reductants (100 μM) and amino acids (100 μM), in phosphate buffered saline (0.5% DMSO) at pH 7.4. Bars represent the final fluorescence intensity at 470 nm (*I*_470_) after a 12 h incubation. Excitation was effected at 340 nm with the excitation and emission slit widths both set at 5 nm. (d) Time course of changes in fluorescence intensity at 470 nm for 40 min in the presence of sodium dithionite.

To assess the behaviour of conjugate **1** upon activation of the trigger, the fluorescence of **1** as a solution in PBS (phosphate buffered saline) was monitored after the addition of sodium dithionite, as an enzyme free model system without potential interference from NAD(P)H fluorescence.12*f* The addition of sodium dithionite (40 μM) to the solution of **1** (20 μM) in aqueous PBS resulted in rapid appearance of an AIE band centered at 470 nm (Fig. 1a and d). By contrast, the release of the fluorophore in MeOH did not result in a significant fluorescence increase (Fig. 1b), confirming the AIE origin of the fluorescence, as MeOH solvation of the released fluorophore prohibits the AIE effect. Probe **1** demonstrated and excellent selectivity profile, showing a more than 200-fold increase in fluorescence upon the addition of sodium dithionite whilst no or negligible amounts of fluorescence increase could be observed upon the addition of other biologically relevant thiols and amino acids (Fig. 1c). The selective triggering by sodium dithionite was further demonstrated by HPLC analysis, showing the complete conversion of **1** to **8** (Fig. S14†).

### Subcellular localization

**Fig. 2:**
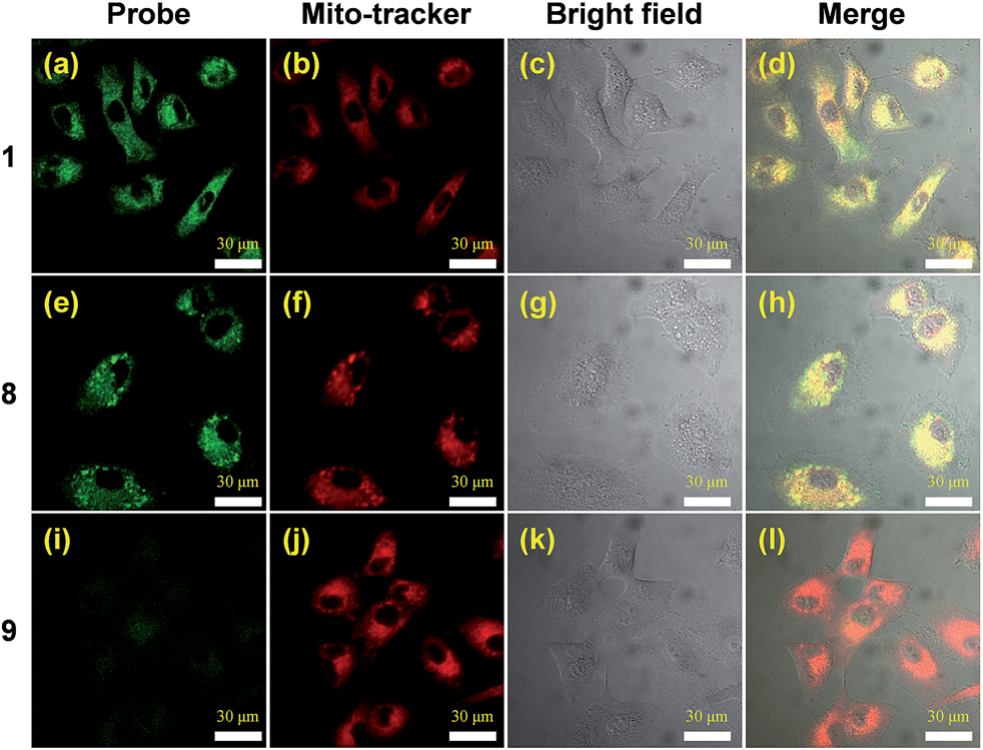
Confocal microscopy images of A549 cells treated with **1**, **8** and **9**. A549 cell after incubation with 20 μM of **1**, **8** and **9** for 12 h, co-stained with 200 nm Mito-tracker deep red. The red fluorescence is from Mito-tracker deep red, *λ*_ex_ = 633 nm, *λ*_em_ = 650–700 nm, and the green fluorescence is from the probes, *λ*_ex_ = 740 nm, *λ*_em_ = 450–550 nm. Cell images were obtained using two photon excitation wavelengths of 740 nm and emission wavelengths of 450–550 nm, green signal, respectively.

The two-photon cross section of probe **8** in water was determined to be 0.47 GM (data not shown), providing a convenient means to visualize the fluorophore by means of two-photon excitation confocal microscopy. Colocalization studies using the NQO1 positive A549 cell line clearly demonstrate the lipophilic delocalized cation's efficiency to bestow mitochondria targeting upon the AIE probe (Fig. 2). Here both triphenylphosphonium bearing compounds **1** and **8** demonstrate efficient intracellular AIE emission originating from the mitochondria (Fig. 2a–h), whereas virtually no fluorescence was observed in the absence of this subcellular targeting agent (Fig. 2i–l).

### *In vitro* dependency of cytotoxicity on NQO1

**Fig. 3:**
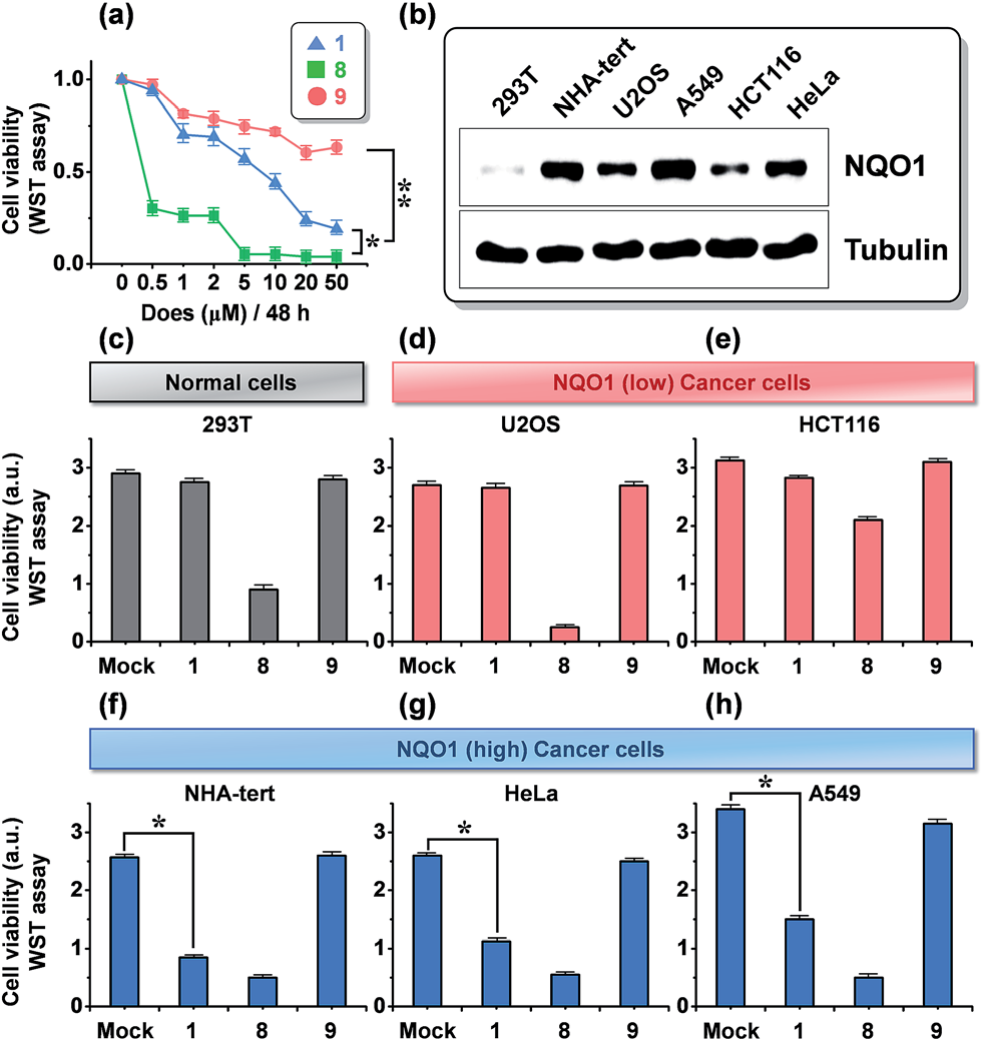
Effect of **1** on tumor cell viability and its association with expression status of NQO1. (a) Cytotoxicity (A549 cells), as determined by a WST-1 assay, after 48 h of incubation with increasing amounts of probes **1**, **8** and **9**. (b) Immunoblot assay showing NQO1 expression levels in six human cells tested in this study. (c–h) Three NQO1 low-expressing cells (c–e), including 293T embryonic kidney cells and three NQO1 high-expressing cells (f–h) were treated with 20 μM of **1**, **8**, and **9** for 48 h. Cell viability was determined using aWST-1 assay. *P < 0.005; **P < 0.001; cell viability data represent the mean and SD of triplicate determinations from a single experiment that has been repeated one or two times with similar outcomes.

The dose dependence of the cytotoxicity of **1**, **8** and **9** in A549 cells, expressing high levels of the NQO1 enzyme, was determined using a WST-1 assay. In contrast to the non-targeted reference compound **9**, triphenylphosphonium appended molecules **1** and **8** showed elevated cytotoxicity (Fig. 3a), which reflects a conclusion in line with the subcellular localization studies reported in Fig. 2. To address whether **1** provokes a NQO1-associated cytotoxic effect, we performed cell viability assays using human embryonic 293T cells and five human cancer cell lines of various tissue origins, which express differential levels of NQO1 protein (Fig. 3b) at a 20 μM dose of probes **1**, **8** and **9**. Intriguingly, treatment of **1** resulted in no or only slightly decreased viability of 293T (normal kidney), U2O3 (osteosarcoma), and HCT116 (colon cancer) cells (Fig. 3c–e), which have low NQO1 expression levels, whereas it caused a considerable decrease in cell viability of NHA (glioma), HeLa (cervical cancer), and A549 (lung cancer) cells, associated with high NQO1 expression levels (Fig. 3f–h). As anticipated, **8** displayed a strong cytotoxic effect on all cell types we tested, indiscriminate of NQO1 levels and pathological state, while **9** failed to show any significant effect. Therefore, this finding supports that **1** exerts its cytotoxic effect on human cancer cells in a highly NQO1-dependent fashion.

**Fig. 4:**
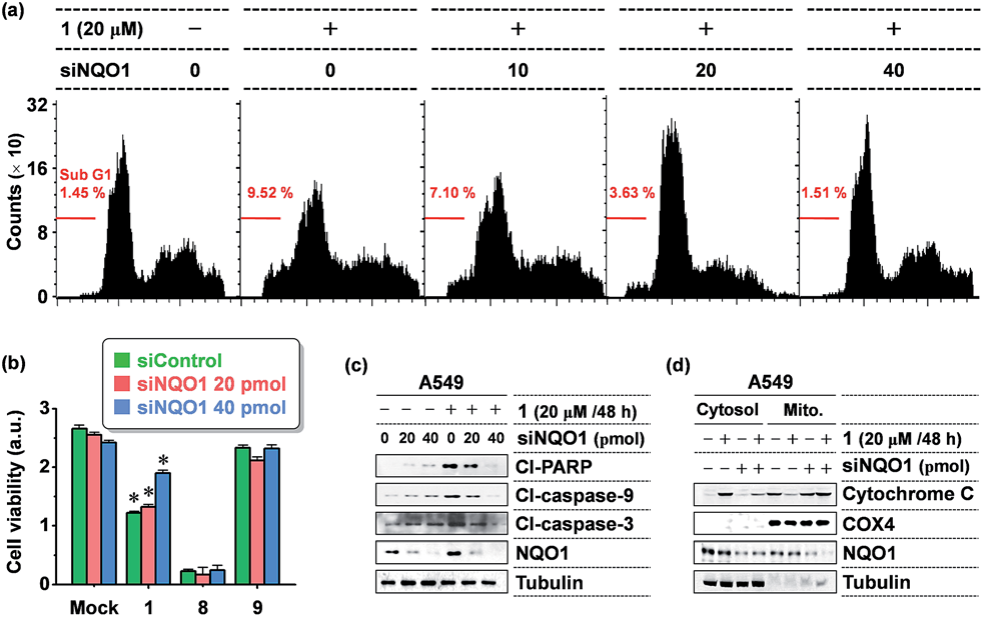
The NQO1-dependency of **1** cytotoxicity. (a) Flow cytometric analysis of apoptosis showing a NQO1 depletion effect on **1** induction of apoptosis. A549 cells transfected with siNQO1 were incubated with **1** as indicated and flow cytometric analysis was carried out to determine the percentages of sub-G1 fraction. (b) Attenuation of **1** cytotoxicity by siRNA-mediated knockdown of NQO1 expression. A549 cells transfected with either siControl or siNQO1 were incubated with **1**, **8**, or **9** (10 M, 24 hours). Cell viability was determined by a WST-1 assay. Each bar represents the average of triplicate measurements; *P < 0.001. (c) NQO1-dependency of **1** activation of apoptosis. Cells transfected with siRNAs were incubated with **1** (20 μM). After 48 hours, an immunoblot assay was performed to measure the cleaved PARP, caspase-3, and caspase-9 levels. Cl: cleaved. (d) NQO1-dependency of **1** on stimulation of cytochrome C release. Cells were transfected with siRNAs and treated **1** as indicated, and lysates from the cytosolic and mitochondrial fractions were subjected to immunoblot assay of cytochrome C. COX4 and tubulin were used as markers for the mitochondria and cytosol fractions, respectively.

To further define the NQO1-dependeny of **1** cytotoxicity, we tested the NQO1 depletion effect on **1** activity in A549 cells, using small interfering RNA (siRNA)-mediated knockdown of NQO1. As shown in Fig. 4b, the viability-decreasing activity of **1** was attenuated by siNQO1 transfection in a dose-dependent manner, while effects of **8** and **9** were not affected by siNQO1. An immunoblot analysis of cleaved PARP and caspases was performed to investigate the induction of apoptosis as a mechanism in the NQO1-dependency on the cytotoxicity of **1**. Probe **1** strongly activated the cleavage of PARP, caspase-9, and caspase-3, and this effect was noticeably blocked in NQO1-depleted cells (Fig. 4c). Consistently, flow cytometric analysis of the apoptotic sub-G1 fraction showed that probe **1**'s induction of apoptosis is suppressed by siNQO1 in a dose-dependent manner (Fig. 4a). In addition, a cell fractionation assay also revealed that **1** stimulates the cytoplasmic release of cytochrome C in a NQO1-dependent manner (Fig. 4d). Together, these results strongly suggest that NQO1-dependent aggregation of **1** within the mitochondria may trigger dysregulation of membrane permeability, leading to cytochrome C release and subsequent caspase activation.

**Fig. 5:**
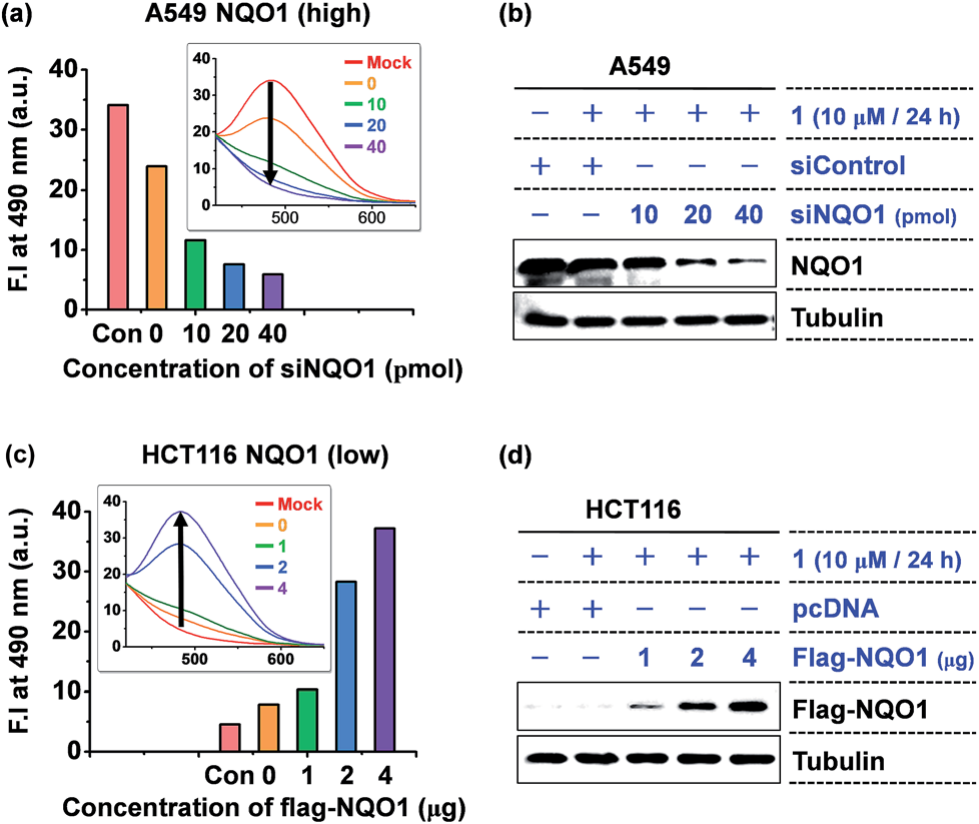
The fluorescence induction of **1** is highly dependent on the NQO1 expression level. (a) A siNQO1 dose-associated decrease in **1**-induced fluorescence intensity in A549 cells. Cells transfected with increasing doses of siNQO1 were incubated with **1** (10 μM). The fluorescence of cell lysates was determined after 24 h incubation. (b) Immunoblot assay of siNQO1-mediated NQO1 knockdown in A549 cells. (c) A NQO1-associated increase in **1**-induced fluorescence intensity in HCT116 cells. Cells transfected with increasing doses of FLAG-NQO1 plasmid were incubated with **1** (10 μM). (d) Immunoblot assay of FLAG-NQO1 levels in HCT116 cells.

Next, the intracellular aggregation of **1** and its dependency on NQO1 was determined by monitoring the relation between the fluorescent intensity of the AIE fluorophore and NQO1 depletion and overexpression. In A549 cells, harboring high NQO1 levels, knockdown of the endogenous NQO1 expression using increasing doses of siNQO1 transfection resulted in a dose-associated reduction in fluorescence intensity (Fig. 5a and b). Consistently, ectopic overexpression of NQO1 using FLAG-NQO1 transfection in HCT116 cells led to a drastic dose-associated increase in the observed AIE fluorescence (Fig. 5c and d). Thus, these results clearly indicate probe **1**'s aggregation and fluorescence induction are highly dependent on the NQO1 expression in tumor cells.

### *In vivo* tumor growth suppression and tumor growth reduction

**Fig. 6:**
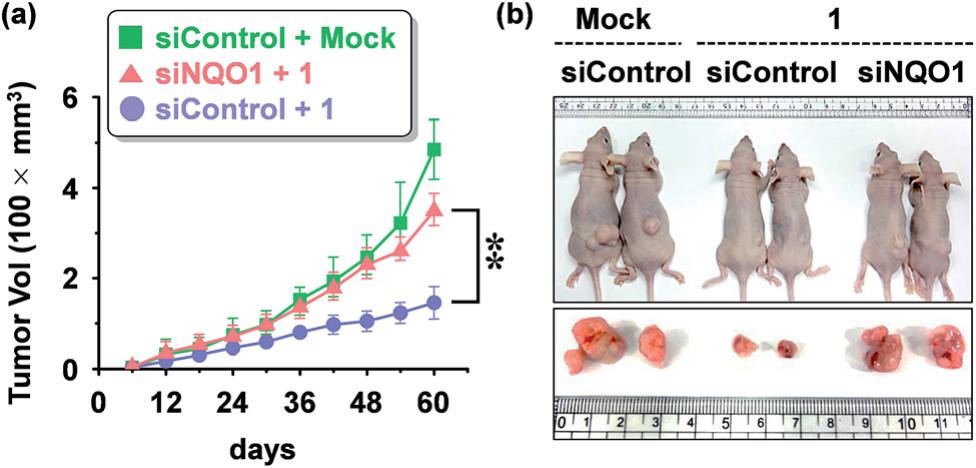
Mouse tumor xenograft assay showing the effect of probe **1** on *in vivo* tumor growth (A549 cells). (a) Inhibition of tumor growth by **1** and its dependency of NQO1. The data represents the mean ± SD (*n* = 5 per group; **P < 0.01). A549 cells transfected with either siNQO1 or siControl were injected subcutaneously in the right flanks of nude mice. **1** was administered by tail vein injection (2 mg kg^–1^ d^–1^) at day **8** and subsequently once per three days for 2 weeks. The tumor growth was monitored periodically, and the volume (*V*) was calculated by using the modified ellipsoidal formula: *V* = 1/2 × length × (width)^2^. (b) Representative photographs of xenograft tumor bearing mice, 5 weeks after administration of **1**.

**Fig. 7:**
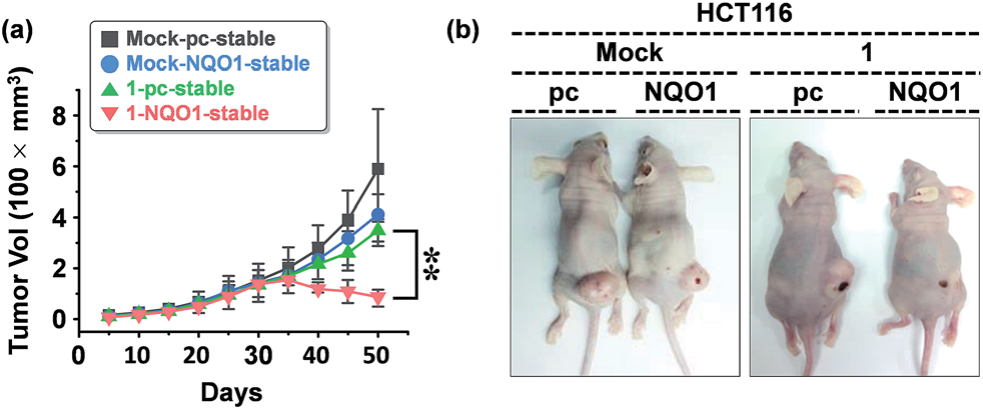
The effect of **1** on the tumor size (HCT116 cells). (a) Growth response of xenograft tumors derived from control (pcDNA) or NQO1-expressing HCT116 cells to prodrug **1** (*n* = 5 per group; error bars indicate ±SD; **P-value < 0.01). HCT116 cells transfected with either pcDNA or NQO1 expression plasmid were injected subcutaneously in the right flanks of nude mice. **1** was administered by tail vein injection (4 mg kg^–1^) at day 10 and subsequently once per five days for 2 weeks. The tumor growth was monitored periodically, and the volume (*V*) was calculated by using the modified ellipsoidal formula: *V* = 1/2 × length × (width)^2^. (b) Representative photographs of xenograft tumors at day 30 after probe **1** injection.

We examined the effect of probe **1** on the tumor growth and its dependency on NQO1 expression, using xenograft tumors of A549 cells with and without NQO1 knockdown. Identical numbers of siControl or siNQO1 cells (1 × 10^7^) were injected subcutaneously into the flank of nude mice. Both cells generated visible tumors of similar mass 8 days subsequent to injection, and **1** was administered by tail vein injection (2 mg kg^–1^ d^–1^) once per three days for 2 weeks. The tumor growth was monitored regularly up to 60 days. Compared with siControl tumors, showing a dramatic response to **1** (79% reduction), siNQO1 tumors displayed a greatly attenuated response (28% reduction), indicating that **1** suppresses tumor growth in a highly NQO1-dependent manner *in vivo* (Fig. 6a). Subsequently, to assess the potential of probe **1** to cause tumor size regression in HCT116 cell based tumors, either transfected with a control plasmid (pcDNA) or a NQO1 expression plasmid (1 × 10^7^ cells) were injected subcutaneously in the flank of nude mice. Tumor growth curves clearly implicate the vital importance on both the presence of the NQO1 enzyme and **1** on the efficiency of the tumor size regression, when treated 10 days post tumor xenograft inoculation. After approximately 35 days post tumor xenograft inoculation a clear divergent behavior could be observed, with the control treatments exhibiting unrestricted growth, and only the combination of both the presence of the enzyme and **1** displaying cancer size regression. Treatment thus ultimately resulted in an approximately 4-fold difference in tumor size, 40 days after the start of the treatment (Fig. 7a).

### *In vivo* and *ex vivo* tissue distribution of aggregated **1**

**Fig. 8:**
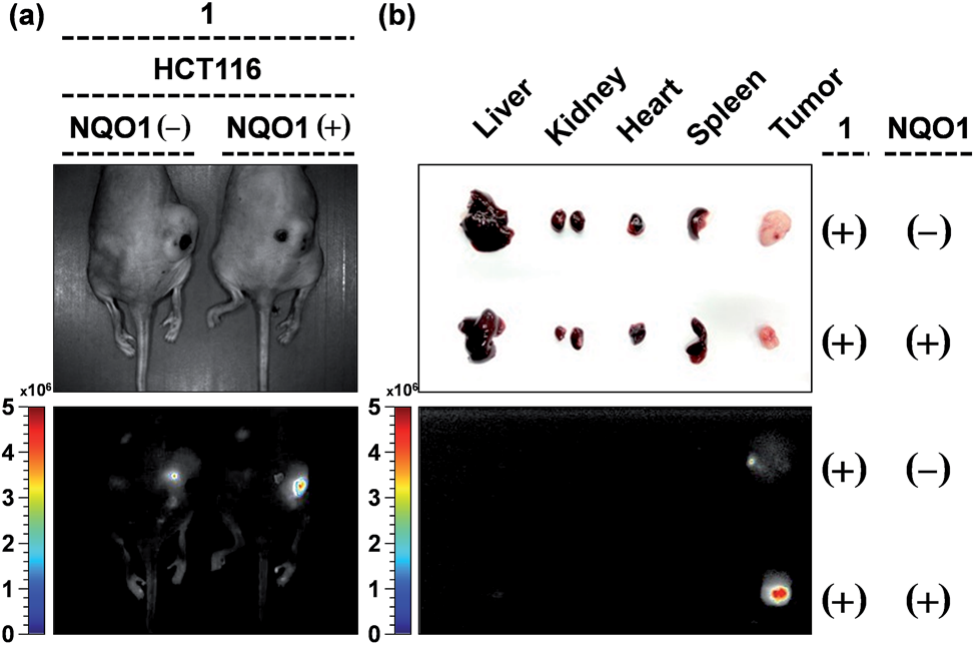
The *in vivo* and *ex vivo* imaging of HCT116 bearing mice 40 days after the start of the treatment (see also Fig. 7). (a) Representative images of mice bearing HCT116 cells, pretreated with control plasmid (left) and NQO1 expression plasmid (right) under white light (top) and the representative *in vivo* fluorescent images of probe **1** accumulation in xenograft tumors (*λ*_ex_ = 430–480 nm, *λ*_em_ = 490–550 nm). (b) Dissected organs and tumors (up) and their fluorescent images (down) of probe **1**-injected mice (*λ*_ex_ = 430–480 nm, *λ*_em_ = 490–550 nm).

Finally, to prove the result of the cancer growth curves was caused by the specific delivery and unique activation of probe **1** in NQO1 positive tumors, the mice bearing HCT116 cells, with and without expression of NQO1 (see also Fig. 7b) were subjected to *in vivo* fluorescence studies 25 days after inoculation with HCT116 cells and 10 days after the start of probe **1** based therapy. As can clearly be seen in Fig. 8a, the probe was specifically upconcentrated in the tumor tissue, and the activation in the case of the NQO1 positive cells, clearly resulted in a higher degree of probe fluorescence and thus *in situ* aggregation. The same mice were subsequently euthanized and the fluorescence of the excised organ tissues was recorded, confirming the superior fluorescence originating from the NQO1 positive cell based tumor. The excellent tissue selectivity, showing no or non-significant levels of fluorescence originating from either the reticulo-endothelial system (liver and spleen) or the renal system (kidneys) as well as the heart, provides further evidence towards the excellent tissue specific and enzyme selective activation of probe **1**.

## Experimental section

### General synthetic methods

The reagents used in this study were purchase from Alfa-Aesar, Aldrich, TCI, Carbosynth, Duksan, and Acros and used without further purification. Silica gel 60 (Merck, 0.040–0.063 mm) was used for column chromatography and Merck 60 F254 silica gel plates were used for analytical thin-layer chromatography. ^1^H and ^13^C NMR spectra were recorded in CDCl_3_ on a Varian 400 MHz instrument. All chemical shifts are reported in ppm values using the peak of TMS as an internal reference. Reverse-phase HPLC experiments were conducted using an Young Lin HPLC system (YL9100) equipped with a VDSpher 100 C18-E column (5 μm, 250 × 4.6 mm) operated at a flow rates of 1 mL min^–1^ using a mobile phase consisting of a binary gradient of solvent A (water with 0.5% v/v TFA) and solvent B (acetonitrile with 0.5% v/v TFA). ESI mass spectrometric analyses were carried out using an LC/MS-2020 Series (Shimadzu) instrument.

### DFT calculations

Density functional theory calculations were performed using B3LYP^15^ at the 6-31G(d) level of theory,^16^ using the Gaussian G09W software package.^17^ Gaussian input file generation and molecular orbital visualization were performed using the Gabedit 2.4.8 software package.^18^ The structure optimization was performed from several starting geometries to ensure a global minimum was reached.

### Human normal and cancer cell lines

Human cancer cell lines and the embryonic kidney 293T cells were purchased from American Type Culture Collection (Rockville, MD, USA) or Korea Cell Line Bank (Seoul, South Korea). The cells were maintained in Dulbecco's modified Eagle's medium supplemented with 10% FBS (GIBCO BRL) at 37 °C in a humidified atmosphere with 5% CO_2_.

### Expression plasmids, siRNA, and transfection

The NQO1 expression vector was purchased from Addgene (MA, USA). Transfections were performed using Lipofectamine 2000 (Invitrogen). siRNA duplex against NQO1 (5′-GAGAGTTTGCTTACACTTA-3′) was synthesized by Dharmacon Research. Transfection of siRNA was performed using a siRNA–Oligofectamine mixture or electroporation (Neon transfection system; Invitrogen).

### Immunoblotting assay

Immunoblot analyses were performed using antibodies specific for NQO1 (SC-16464), cleaved PARP (5625), cleaved caspase-3 (9664), cleaved caspase-9 (7237), COX-4 (4850), and cytochrome C (4280), purchased from Santa Cruz Biotechnology or Cell Signaling Technology. Anti-Flag (F3165) and anti-tubulin antibodies were obtained from Sigma and Invitrogen, respectively. Briefly, cells were washed twice in ice-cold PBS and lysed in a radioimmunoprecipitation assay buffer containing 50 mM Tris·HCl (pH 7.5), 150 mM NaCl, 0.1% SDS, 1% Triton X-100, 1% sodium deoxycholate, and protease inhibitor mixture. After sonification, the lysate was centrifuged, and supernatant was recovered and loaded on an 8% SDS-polyacrylamide gel for electrophoresis.

### Flow cytometric analysis of apoptosis

Cells transfected with siNQO1 or siControl were treated with **1** (20 μM). After 24 h treatment, the cells were fixed with 70% ethanol and resuspended in 1 mL of PBS containing 50 mg mL^–1^ RNase and 50 mg mL^–1^ propidium iodide (Sigma). The assay was performed on a FACScan flow cytometer (Becton Dickinson, San Jose, CA) and the apoptotic profile was analyzed using Modfit software (Becton Dickinson).

### Separation of cytosolic and mitochondria fractions

Separation of cytosolic and mitochondria fractions was performed using the Mitochondria Isolation Kit (Thermo Scientific, USA). Briefly, the cells were washed with cold PBS and lysed by 5-cycle freeze-thawing in Mitochondria Isolation Reagent A buffer, containing 10 mM phenylmethylsulfonyl fluoride (PMSF). After ice incubation for 10 min and Mitochondria Isolation Reagent B buffer addition, the lysates were centrifuged at 700*g* for 10 min following inverting after treatment of the Mitochondria Isolation Reagent C buffer. The supernatant was transferred to a new tube, centrifuged at 12 000*g* for 15 min at 4 °C, and collected as the cytosolic fraction. After addition of 500 μL of Mitochondria Isolation Reagent C, the pellet was centrifuged at 12 000*g* for 5 min and collected as the mitochondrial fraction.

### Animal studies

Four-week-old immunodeficient nude mice (nu/nu) mice (Orient Bio Inc.) were maintained in pressurized ventilated cages. Cells transfected with siNQO1 or siControl (1 × 10^7^) were injected subcutaneously into five mice. **1** was administered by tail vein injection (2 mg kg^–1^ d^–1^) for once per three days for 2 weeks. HCT116 cells (1.0 × 10^7^) transfected with empty (pcDNA) or NQO1 expression plasmid were injected subcutaneously into the right flank of mice. When tumor growth reached a detectable size (approx. 20 mm^3^), the mice were treated with compound **1** (4 mg kg^–1^) or vehicle PBS by tail vein injection once every 5 days for 20 days. Tumor growth was monitored periodically, and volume (*V*) was calculated by using the modified ellipsoidal formula: *V* = 1/2 × length × (width)^2^. All animal studies were performed with the approval of Korea University Institutional Animal Care and Use Committee and Korea Animal Protection Law.

### *In vivo* and *ex vivo* fluorescent imaging

To study tumor target specificity and organ distribution of probe **1***in vivo* and *ex vivo*, stably expressed empty (pcDNA) or NQO1 HCT116 cells were injected subcutaneously into the mice. When tumor growth reached a detectable size (approx. 20 mm^3^), the mice were treated with probe **1** (4 mg kg^–1^) or vehicle PBS by tail vein injection once every 5 days for 20 days. After 20 days of probe **1** injections, mouse were sacrificed and *in vivo* fluorescence intensity of the tumors and organs of the control and injected mice were measured using Maestro2 (excitation and emission; 500 and 650 nm, respectively). The fluorescence images and autofluorescence were then unmixed with commercial software (Maestro software ver. 2.4, CRi, Woburn, MA, USA) using the multiexcitation spectral analysis function.

### Synthesis of **1**

EDC·HCl (41 mg, 0.26 mmol) was added to a solution of **3** (100 mg, 0.13 mmol), **5** (45 mg, 0.12 mmol) and DMAP (32 mg, 0.26 mmol) in CH_2_Cl_2_ (20 mL) at room temperature. After 12 h the mixture was washed with an aqueous 1 N HCl solution, the organic layer was dried with MgSO_4_, filtered and concentrated *in vacuo*. Purification by column chromatography yielded 114 mg (78% yield) of the title product. ^1^H NMR (CDCl_3_, 400 MHz): 7.86–7.75 (m, 9H), 7.70–7.65 (m, 5H), 7.37–7.19 (m, 7H), 7.17–7.01 (m, 16H), 6.96–6.88 (m, 1H), 3.88–3.70 (m, 3H), 3.03 (d, *J* = 4.76 Hz, 2H), 2.27 (q, *J* = 7.28 Hz, 2H), 2.10 (d, *J* = 18.36 Hz, 3H), 1.87–1.80 (m, 6H), 1.76–1.47 (m, 8H), 1.36 (s, 3H), 1.27 (s, 3H), 0.96–0.83 (m, 3H) ppm. ^13^C NMR (CDCl_3_, 100 MHz): *δ* 12.26, 12.29, 12.83, 12.86, 14.34, 14.43, 14.49, 23.83, 24.07, 24.21, 24.23, 25.46, 28.72, 28.83, 29.66, 29.78, 29.83, 29.95, 31.76, 33.67, 33.70, 34.84, 38.07, 38.12, 47.57, 47.65, 53.75, 118.01, 118.06, 118.86, 118.92, 123.94, 123.06, 123.09, 128.45, 126.53, 126.62, 126.76, 126.78, 126.87, 127.87, 127.91, 128.00, 128.27, 128.33, 128.39, 128.55, 130.65, 130.77, 130.83, 130.89, 131.00, 131.06, 131.42, 131.46, 131.47, 131.53, 131.55, 131.59, 131.62, 131.66, 133.75, 133.78, 133.85, 133.88, 134.53, 134.59, 134.64, 134.68, 153.23, 135.23, 135.24, 135.26, 135.55, 135.57, 135.66, 135.77, 138.44, 138.48, 138.72, 138.74, 140.89, 140.93, 140.95, 142.92, 142.98, 143.04, 143.07, 143.11, 143.15, 143.71, 143.80, 147.59, 147.60, 147.84, 147.85, 152.41, 152.46, 171.59, 171.64, 171.93, 171.99, 187.55, 187.58, 190.91, 190.96 ppm. ESI-MS: *m*/*z* calcd for C_76_H_68_O_6_P (M + H) 1107.47; detected 1107.25.

### Synthesis of **2**

A solution of **6** (109 mg, 0.43 mmol) in DCM was slowly added dropwise to a solution of EDC·HCl (180 mg, 1.16 mmol), **3** (300 mg, 0.58 mmol), and DMAP (141 mg, 1.16 mmol) in CH_2_Cl_2_ (30 mL) at 0 °C. After 12 h the mixture was washed with 1 N aqueous HCl, the organic layer was dried with MgSO_4_, filtered and concentrated *in vacuo*. Purification by column chromatography yielded 243 mg of the title product (56% yield). ^1^H NMR (CDCl_3_, 400 MHz): *δ* 7.35–7.30 (m, 4H), 7.29–7.18 (m, 6H), 7.17–7.08 (m, 13H), 6.98–6.91 (m, 3H), 3.04 (d, *J* = 6.24 Hz, 2H), 2.12 (d, *J* = 15.12 Hz, 3H), 1.86 (t, *J* = 14.04 Hz, 6H), 1.38 (s, 3H), 1.30 (s, 3H) ppm. ^13^C NMR (CDCl_3_, 100 MHz): 12.32, 12.35, 12.88, 12.92, 14.41, 14.52, 14.56, 23.92, 28.77, 28.89, 31.85, 38.14, 38.18, 116.04, 116.19, 121.00, 121.04, 123.98, 123.02, 126.61, 126.65, 126.87, 127.00, 127.93, 127.96, 128.05, 128.15, 128.43, 128.46, 128.51, 128.58, 128.61, 128.65, 129.25, 129.27, 130.38, 130.47, 131.08, 131.15, 131.54, 131.58, 131.65, 131.69, 131.72, 132.39, 132.42, 134.73, 135.33, 135.55, 135.69, 135.84, 138.60, 138.63, 138.86, 140.91, 140.92, 141.21, 141.30, 143.00, 143.03, 143.23, 143.23, 143.35, 143.52, 143.70, 143.79, 143.84, 147.67, 147.68, 152.51, 152.55, 152.72, 152.76, 171.70, 171.76, 187.68, 191.06 ppm. ESI-MS: *m*/*z* calcd for C_52_H_44_O_5_ (M + Na) was 771.31; detected 771.30, (M + K) 787.28, detected 787.25.

### Synthesis of **3**

To a solution of **4** (1.0 g, 2.05 mmol), 2-hydroxyphenylboronic acid (1.13 g, 8.19 mmol) in DME (40 mL) and 2 M Na_2_CO_3_ 20 mL in a 100 mL two-neck round bottom flask, 24 mg of tetrakis(triphenylphosphine)palladium(0) was added. The mixture was stirred at room temperature for 1 h, and subsequently heated under reflux conditions for 12 h. After completion of the reaction, the mixture was washed with a 1 N HCl solution, the organic layer was dried with MgSO_4_, filtered and concentrated *in vacuo*. The reissue was purified by column chromatography. ^1^H NMR (CDCl_3_, 400 MHz): *δ* 7.25–7.08 (m, 23H), 6.96–6.91 (m, 4H) ppm. ^13^C NMR (100 MHz, CDCl_3_) *δ* 116.09, 121.11, 126.96, 127.93, 128.05, 128.58, 129.32, 130.43, 131.59, 132.42, 135.53, 141.17, 143.41, 143.52, 152.60 ppm. ESI-MS: *m*/*z* calcd for C_38_H_28_O_2_ (M + Na) 539.20, detected 539.25, (M + K) 555.17, detected 555.25.

### Synthesis of **4**

This compound was synthesized, according to a previously reported procedure,^19^ with a 77% yield.

### Synthesis of **5**

This compound was synthesized, according to a previously reported procedure,12*g* with a 98% yield.

### Synthesis of **6**

This compound was synthesized, according to a previously reported procedure,^13^ with a 84% yield.

### Synthesis of **7**

This compound was synthesized, according to a previously reported procedure,^13^ with a 98% yield.

### Synthesis of **8**

EDC·HCl (63 mg, 0.40 mmol) was added to a solution of **2** (152 mg, 0.20 mmol), **5** (68 mg, 0.18 mmol) and DMAP (49 mg, 0.40 mmol) in CH_2_Cl_2_ (20 mL) at room temperature. After 12 h the mixture was washed with an aqueous 1 N HCl solution, the organic layer was dried with MgSO_4_, filtered and concentrated *in vacuo*. Purification by column chromatography yielded 160 mg (72%) of the title compound. ^1^H NMR (CDCl_3_, 400 MHz): *δ* 7.76–7.57 (m, 15H), 7.50 (d, *J* = 7.88 Hz, 1H), 7.44 (d, *J* = 8.24 Hz, 1H), 7.32–7.21 (m, 6H), 7.10–6.96 (m, 17H), 6.82–6.69 (m, 2H), 3.73–3.46 (m, 3H), 2.20 (t, *J* = 7 Hz, 1H), 2.02 (t, *J* = 6.96 Hz, 1H), 1.61–1.26 (m, 10H), 0.97–0.83 (m, 3H) ppm. ^13^C NMR (CDCl_3_, 100 MHz): 14.37, 23.87, 24.15, 31.80, 33.75, 34.88, 117.47, 117.92, 117.96, 118.77, 118.81, 119.28, 123.94, 126.37, 126.65, 127.46, 127.83, 127.89, 128.44, 128.69, 128.71, 130.33, 130.64, 130.67, 130.77, 130.80, 131.17, 131.40, 131.62, 131.66, 133.70, 133.73, 133.80, 133.83, 134.79, 135.23, 135.26, 135.63, 136.87, 137.41, 140.35, 140.64, 141.46, 141.76, 142.09, 143.26, 143.99, 144.04, 147.90, 154.44, 155.49, 171.96, 172.33 ppm. ESI-MS: *m*/*z* calcd for C_62_H_52_O_3_P (M + H) was 875.36; detected 875.35.

### Synthesis of **9**

EDC·HCl (60 mg, 0.38 mmol) was added to a solution of compound **3** (100 mg, 0.19 mmol), **6** (145 mg, 0.58 mmol) and DMAP (47 mg, 0.38 mmol) in CH_2_Cl_2_ (30 mL) at room temperature. After 12 h the mixture was washed with an aqueous 1 N HCl solution, the organic layer was dried with MgSO_4_, filtered and concentrated *in vacuo*. Purification by column chromatography yielded 118 mg (82% yield) of the title compound. ^1^H NMR (CDCl_3_, 400 MHz): *δ* 7.85–6.91 (m, 26H), 3.28 (s, 4H), 2.15 (s, 6H), 1.90–1.89 (m, 12H), 1.51 (s, 12H). ^13^C NMR (CDCl_3_, 100 MHz): 12.34, 12.86, 14.59, 28.96, 38.27, 47.45, 123.61, 123.62, 126.95, 138.76, 139.21, 142.20, 143.09, 152.13, 170.76, 187.63, 191.09 ppm. ESI-MS: *m*/*z* calcd for C_66_H_60_O_8_ (M + Na) was 1003.42; detected 1003.45, (M + K) was 1019.39; detected 1019.35.

## Conclusions

In summary, we hereby report a selective NQO1 dependent dual cancer and mitochondria targeting AIE fluorophore **1**. Importantly, it was shown that both the mitochondrial localization of the probe and the presence of a NQO1 cleavable linker was essential to the pathology specific toxicity. The crucial dependence of the self-aggregation process and the associated cytotoxicity cleaved **1** on the expression levels of NQO1 was further demonstrated both *in vitro* and *in vivo* by NQO1 expression modulation. The cytotoxicity was determined to originate from the caspase-dependent induction of apoptosis subsequent to cytochrome C release by membrane permeabilization. Probe **1** demonstrated an *in vivo* tumor growth suppression of 79%, a result highly dependent on the expression of NQO1, as gene knockdown resulted in a significantly attenuated growth inhibition effect (28% growth reduction) in A549 tumor xenografts. The tumor size regression at 4 mg kg^–1^ every five days also clearly demonstrated the efficiency of the probe in HCT116 tumor xenografts with observed continuous tumor growth in the control samples and NQO1 absent cell line xenograft and a clear tumor size reduction in the presence of NQO1 expressing tumors and probe **1** treatment. Tissue distribution of the aggregated AIE fluorophores both *in vivo* and *ex vivo* demonstrated a remarkable tumor specificity. The results obtained in this study fully demonstrate the potential of this novel cytotoxic drug, specifically acting on the disruption of mitochondrial function and integrity for NQO1 expressing tumors.

## Supplementary Material

Supplementary information
