# Supplementary material for: Mitochondria-targeted aggregation induced emission theranostics: crucial importance of in situ activation
Source: Chem Sci. 2016 Jun 7;7(9):6050–9. doi: 10.1039/c6sc02236g (PMC6022148; doi:10.1039/c6sc02236g)
Supplement: Supplementary file 1 [file SC-007-C6SC02236G-s001.pdf]

Electronic Supplementary Material (ESI) for Chemical Science.
This journal is © The Royal Society of Chemistry 2016

Supplementary information

Mitochondria-Targeted Aggregation Induced Emission Theranostics: Crucial Importance of In Situ Activation

Weon Sup Shin,[‡a] Min-Goo Lee,[‡b] Peter Verwilst,[‡a] Joung Hae Lee,[c] Sung-Gil Chi,[*b] and Jong Seung Kim,[*a]

a Department of Chemistry, Korea University, Seoul 136-701, Korea.
b School of Life Sciences and Biotechnology, Korea University, Seoul 136-701, Korea
c Korea Research Institute of Standards and Science, Daejeon 305-600, Korea.

‡ These authors contributed equally

Correspondence to: Sung-Gil Chi (chi6302@korea.ac.kr) and Jong Seung Kim (jongskim@korea.ac.kr)

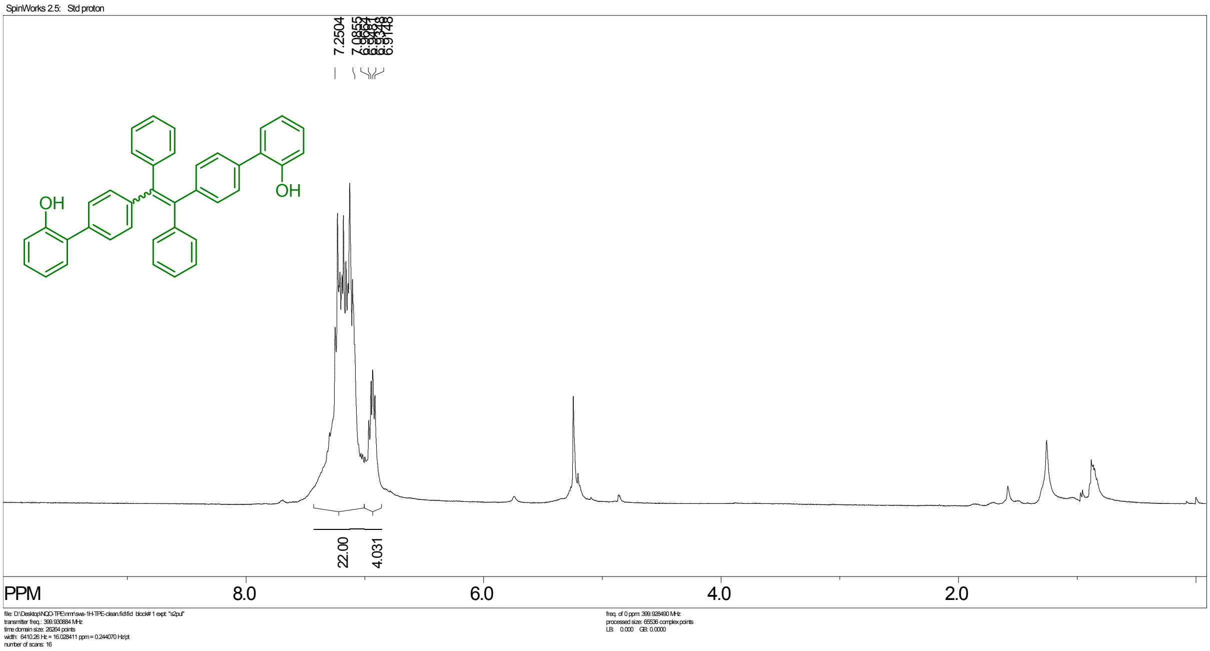

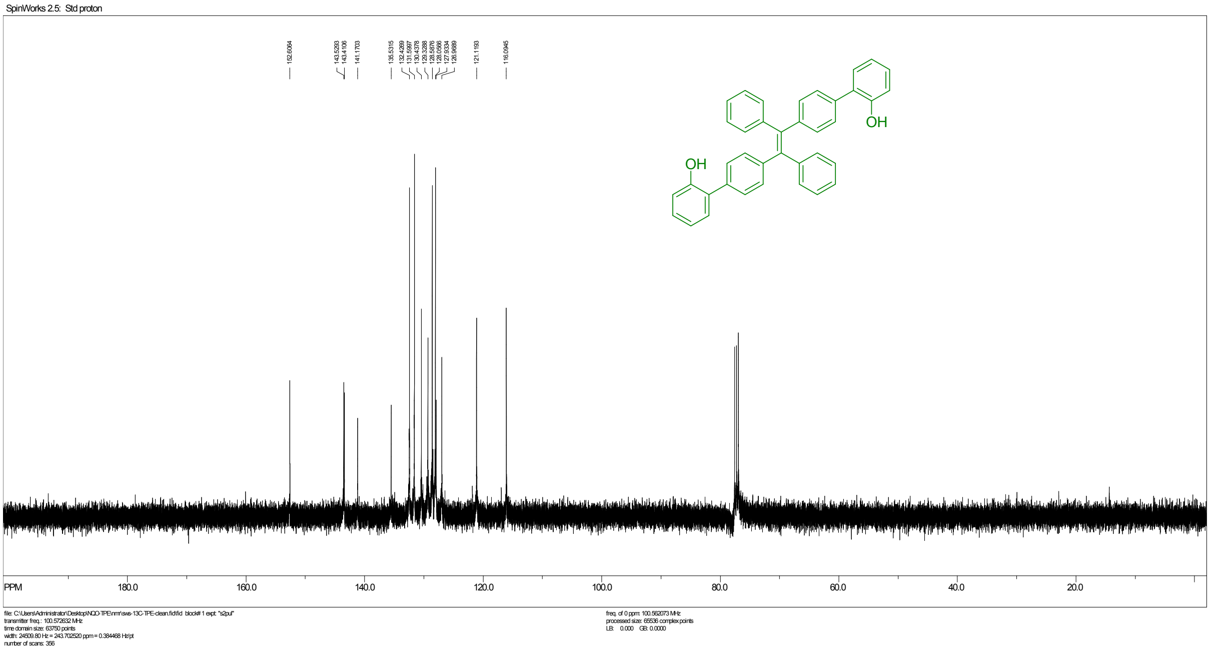

Figure S1: ^{1}H and ^{13}C NMR of **3**.

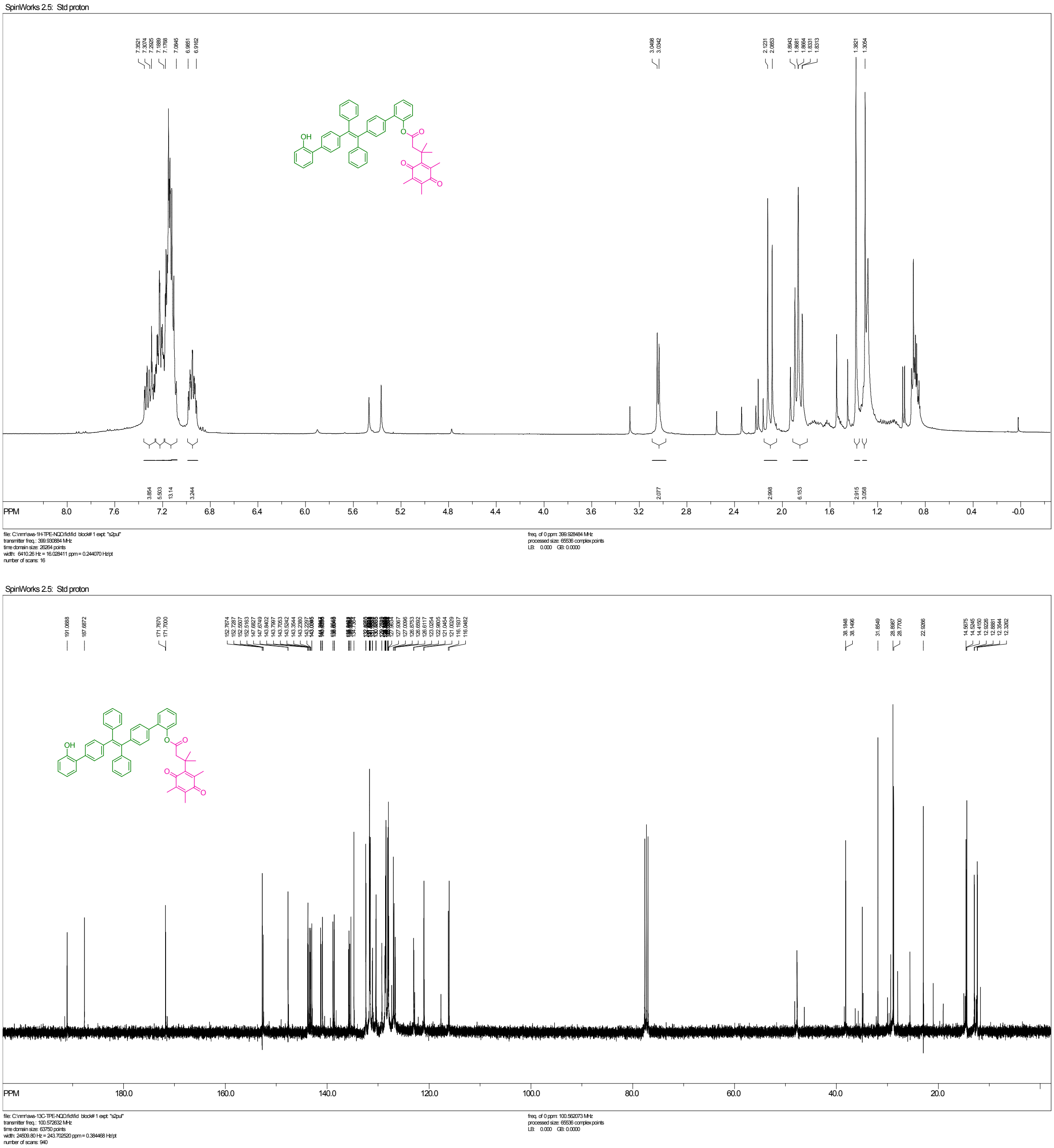

Figure S2: ^{1}H and ^{13}C NMR of **2**.

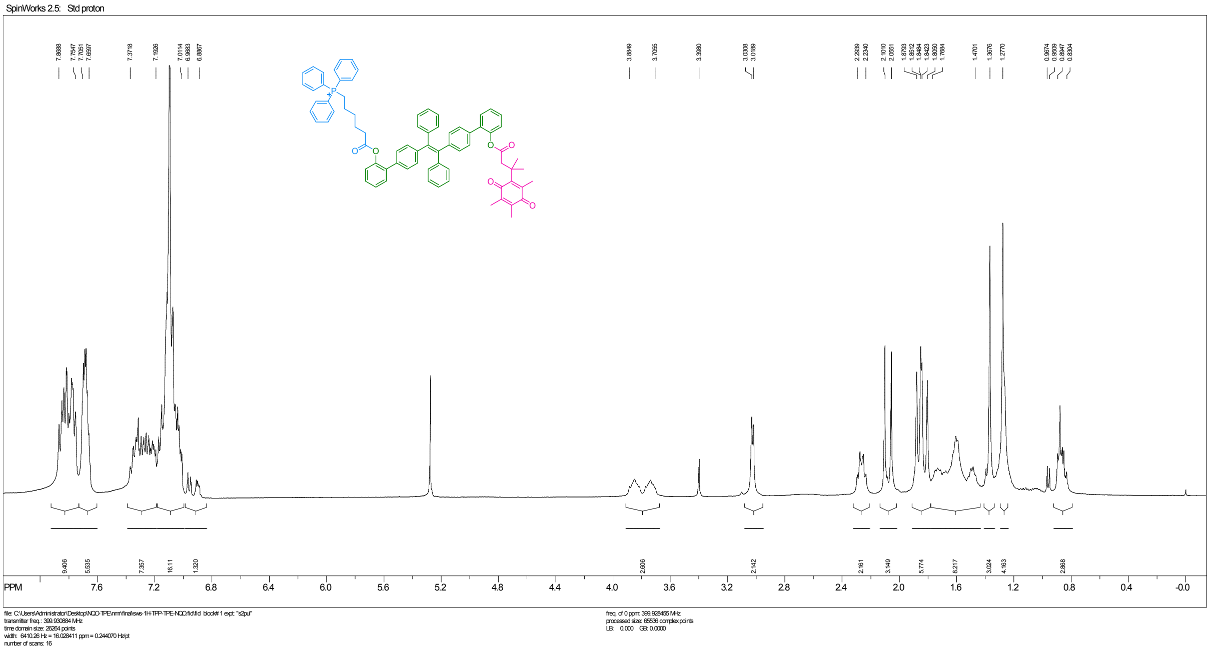

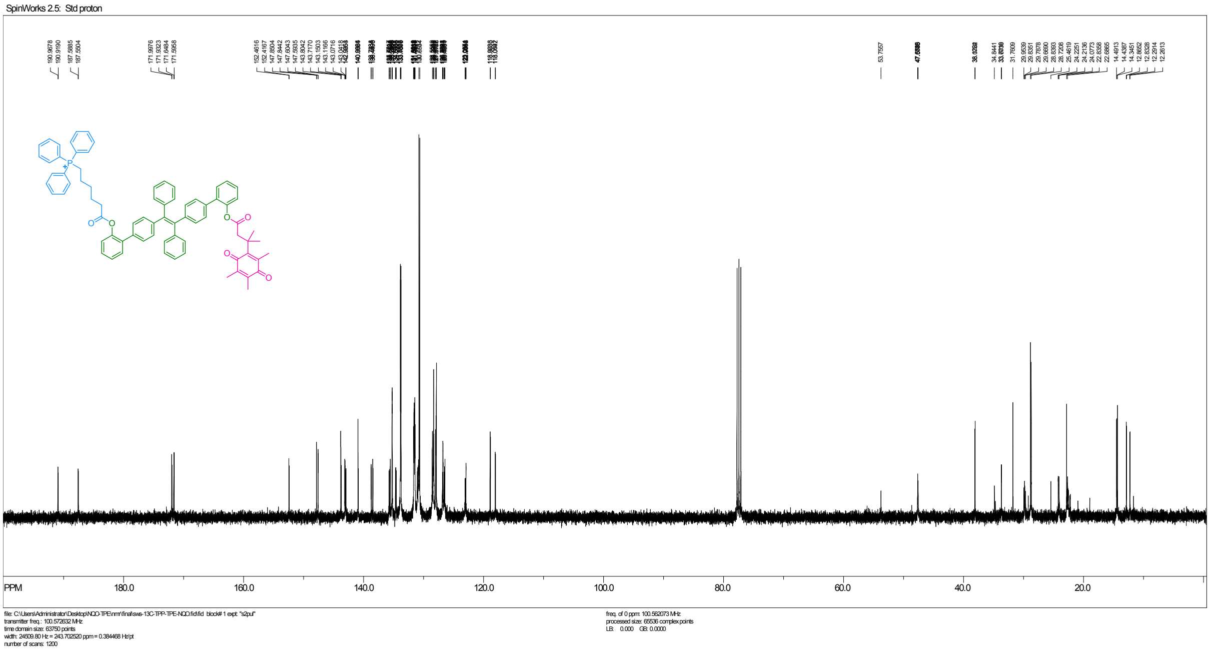

Figure S3: ^{1}H and ^{13}C NMR of **1**.

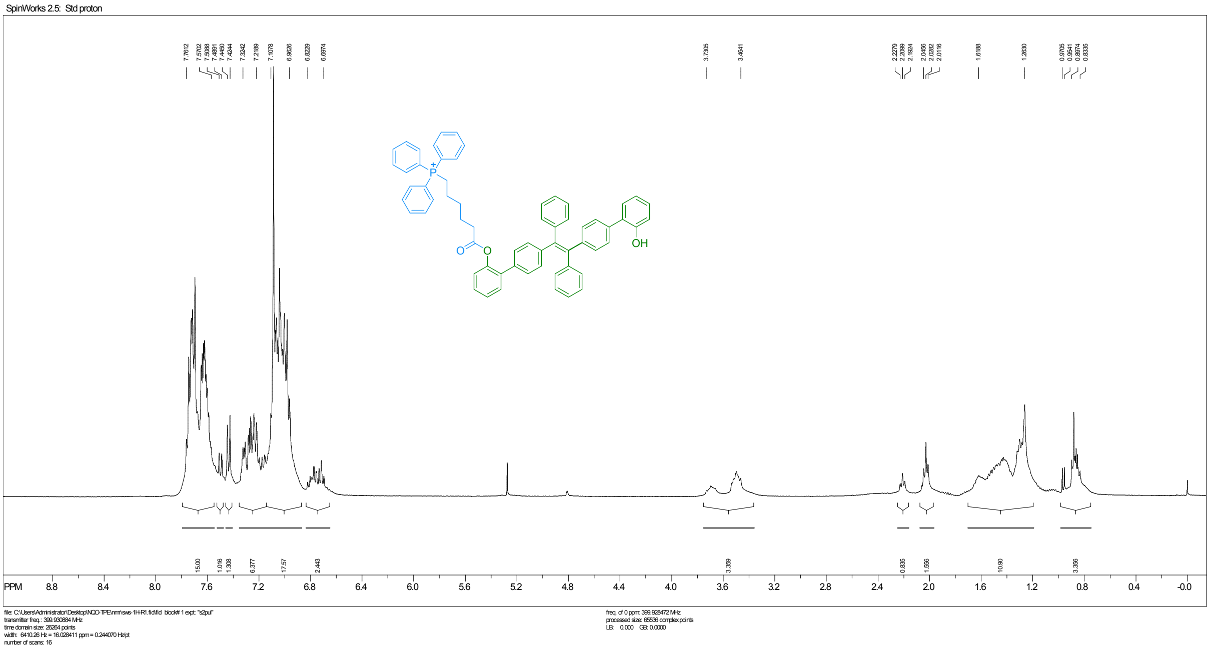

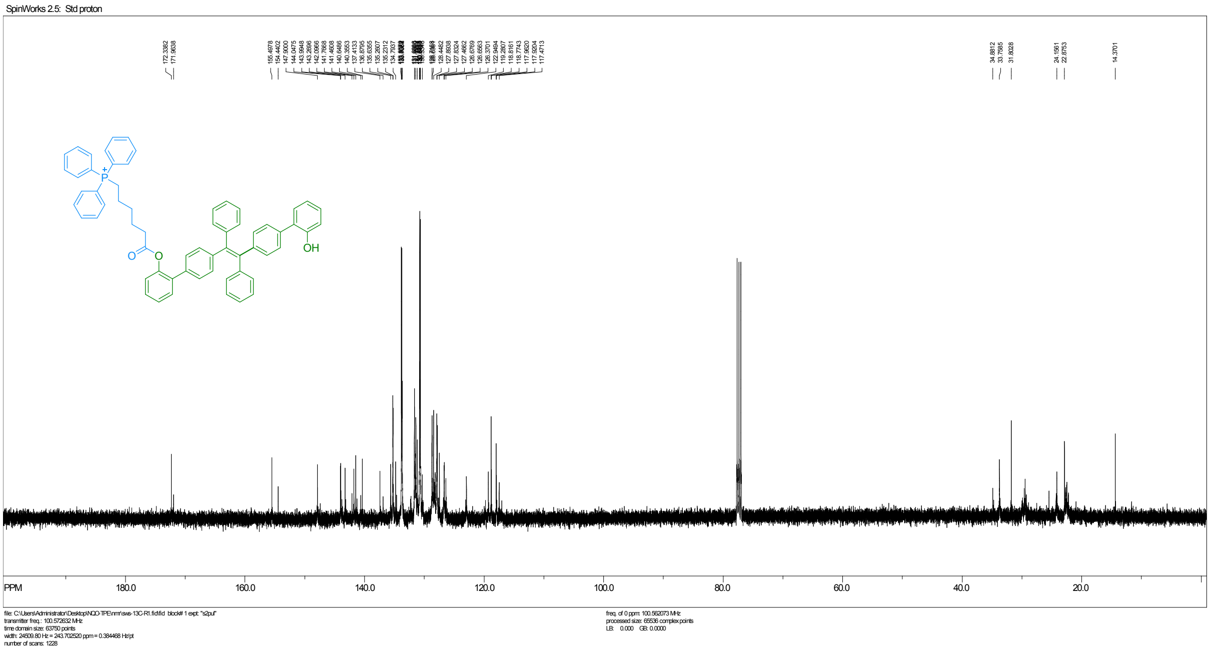

Figure S4: ^{1}H NMR and ^{13}C NMR of **8**.

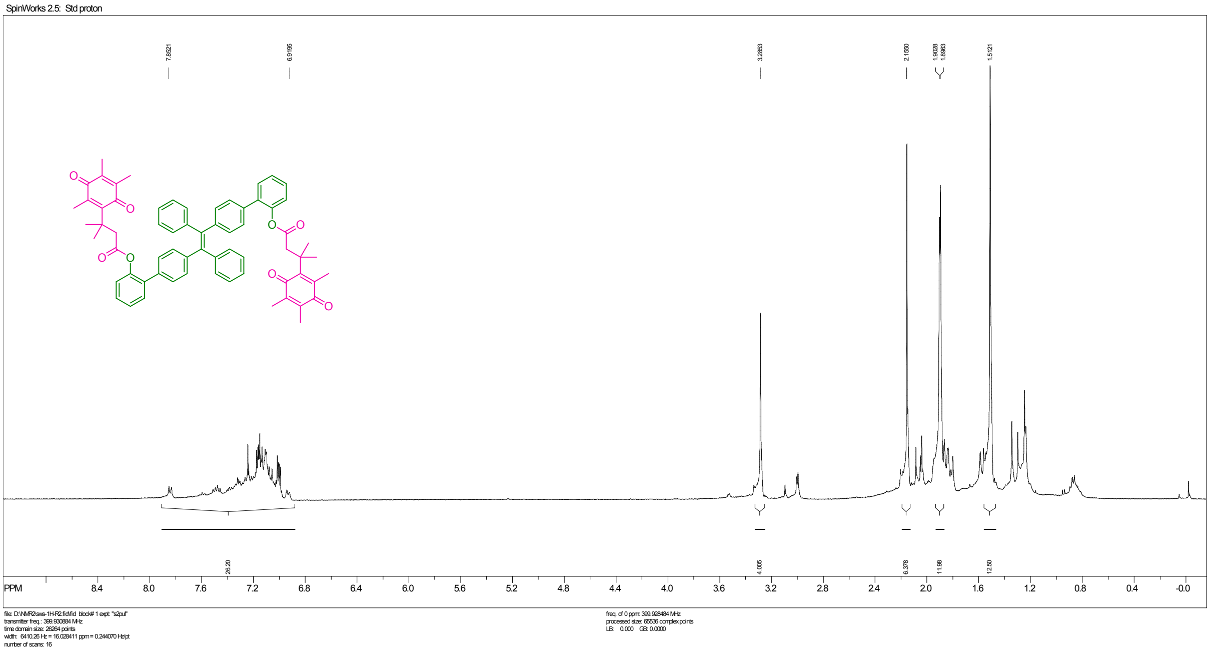

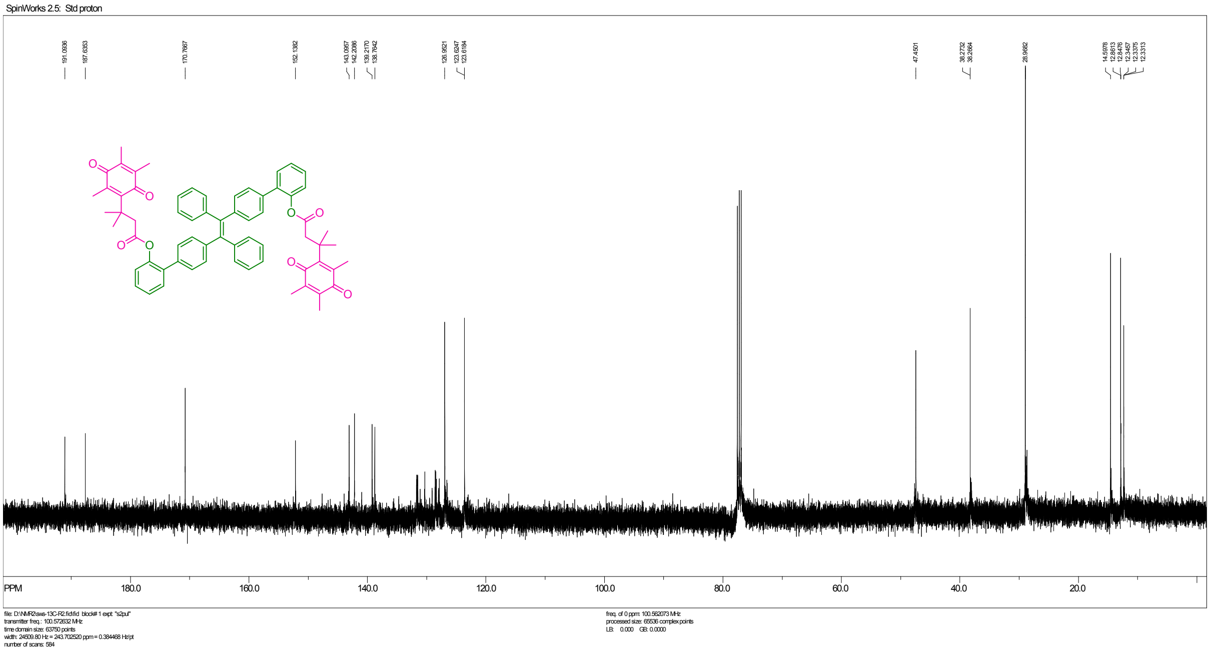

Figure S5: ^{1}H NMR and ^{13}C NMR of **9**.

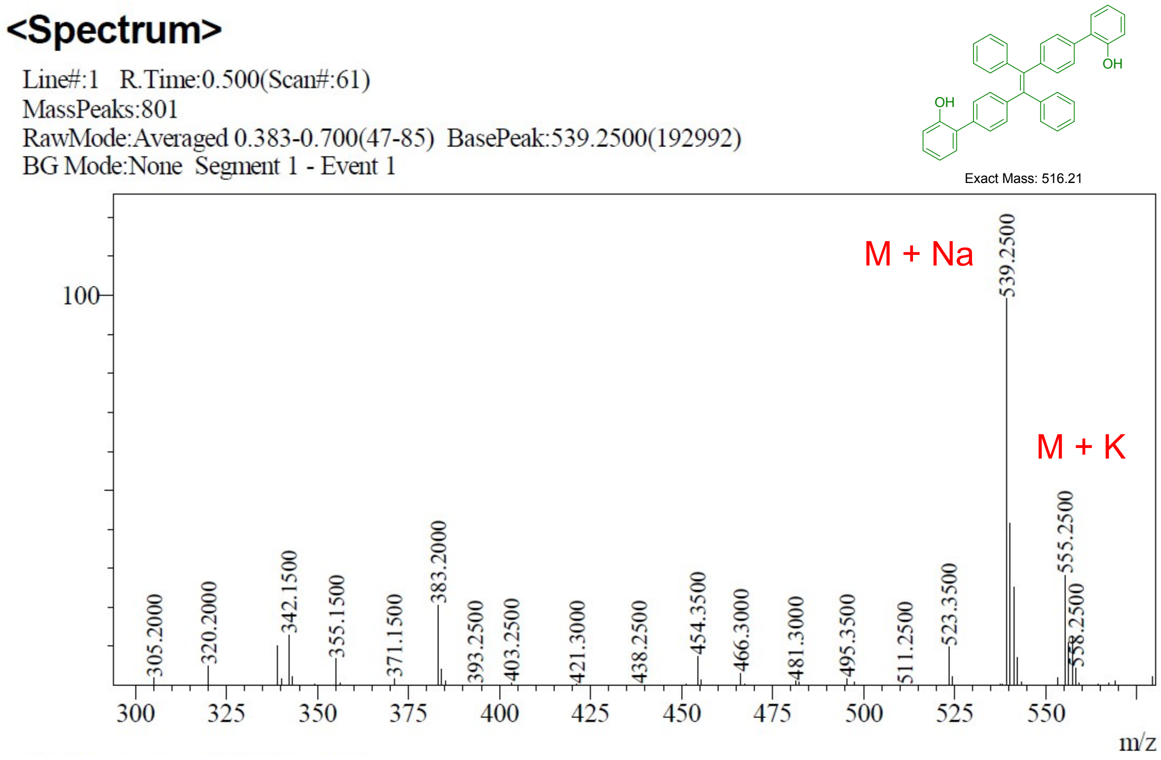

Figure S6: MS data of **3**.

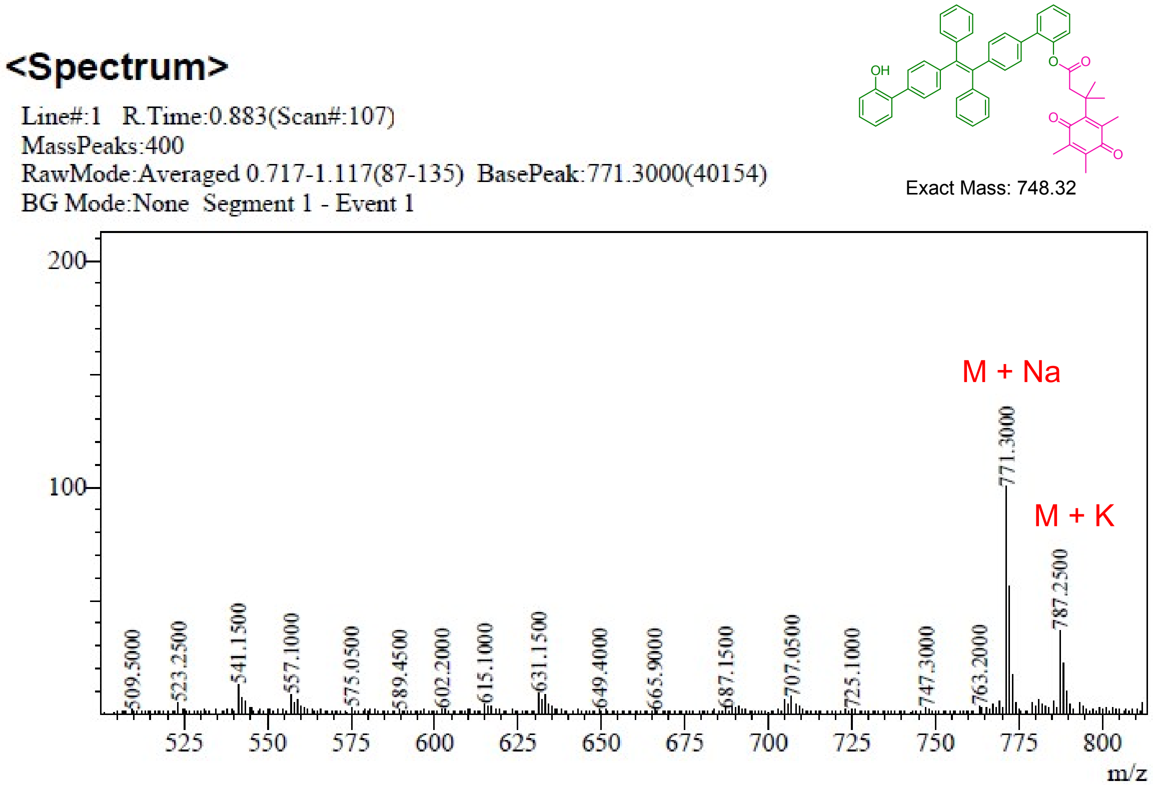

Figure S7: MS data of **2**.

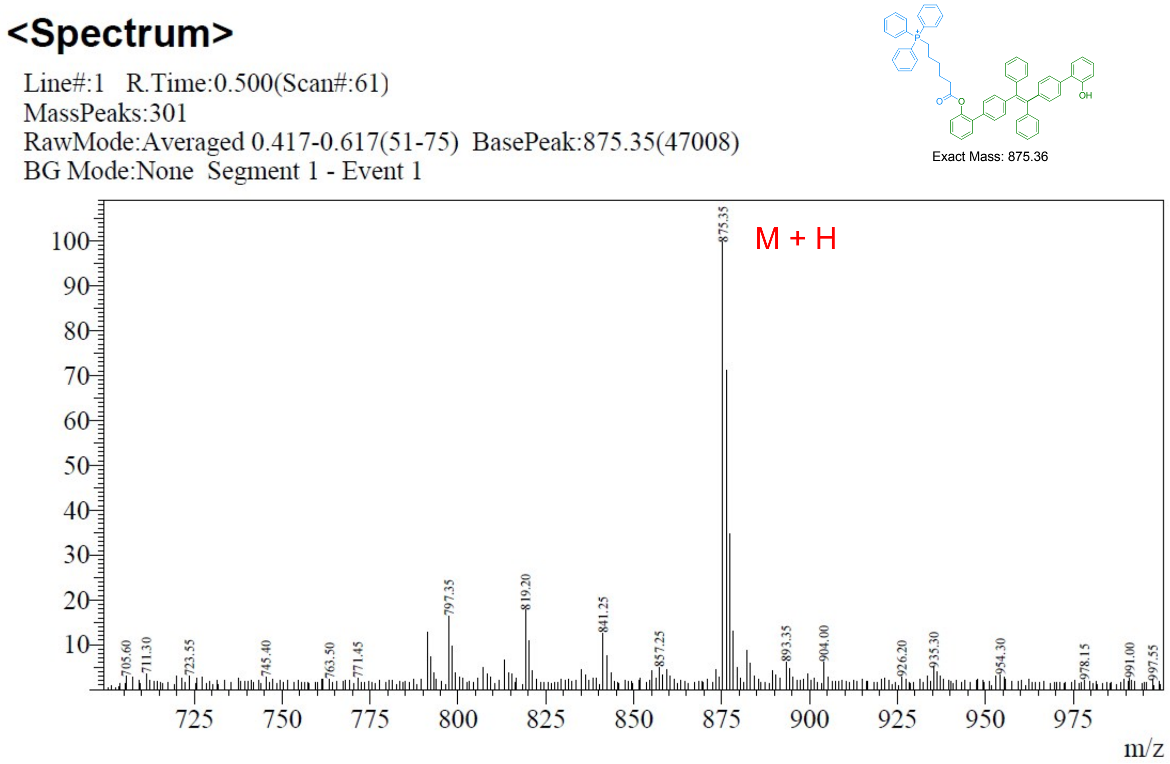

Figure S8: MS data of **8**.

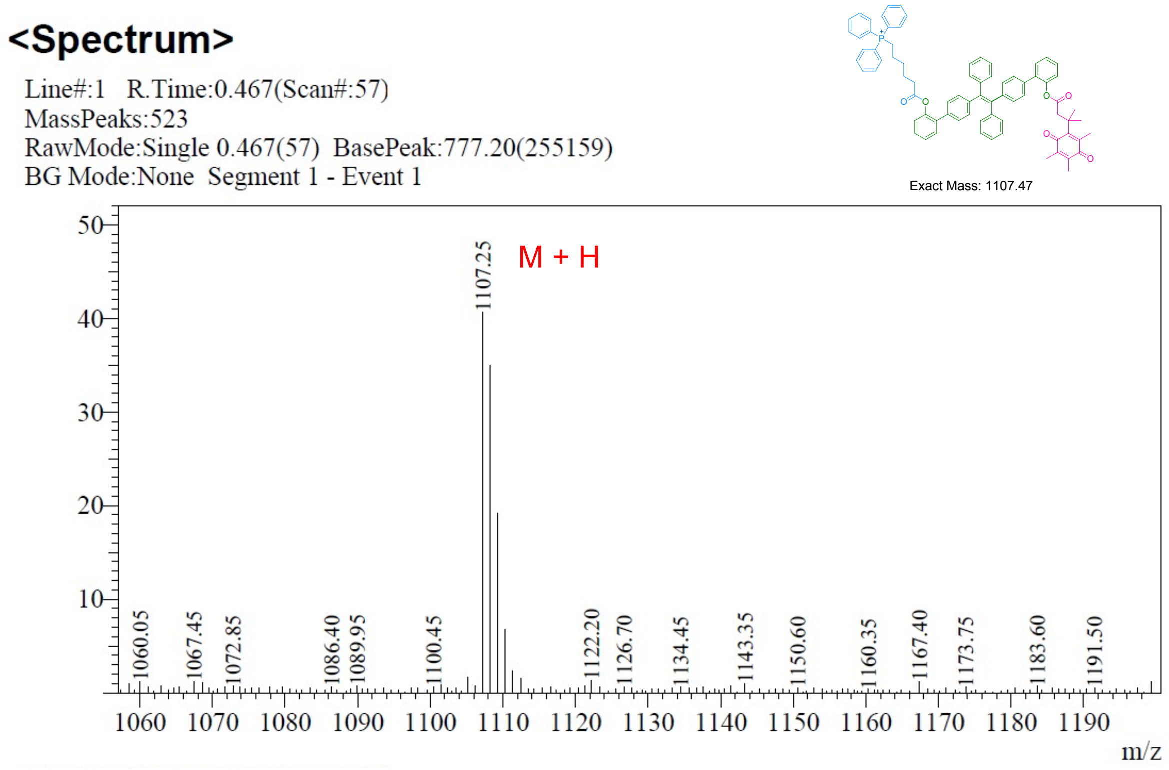

Figure S9: MS data of **1**.

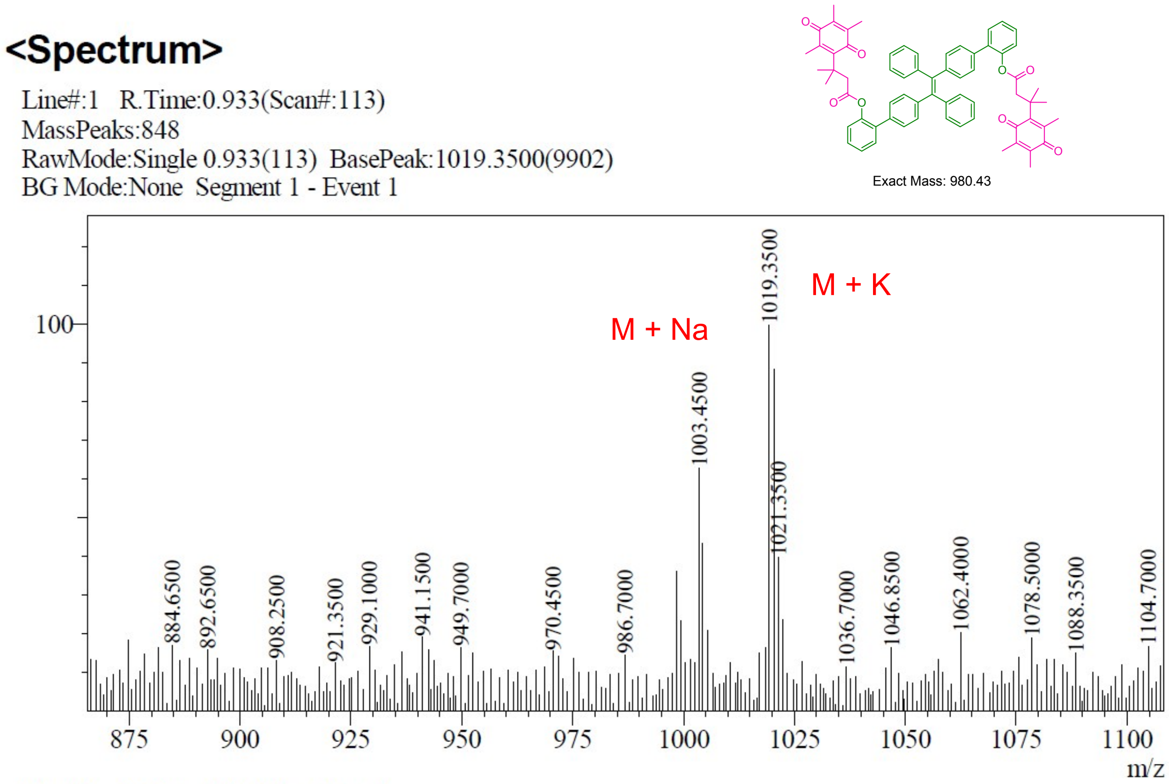

Figure S10: MS data of **9.**

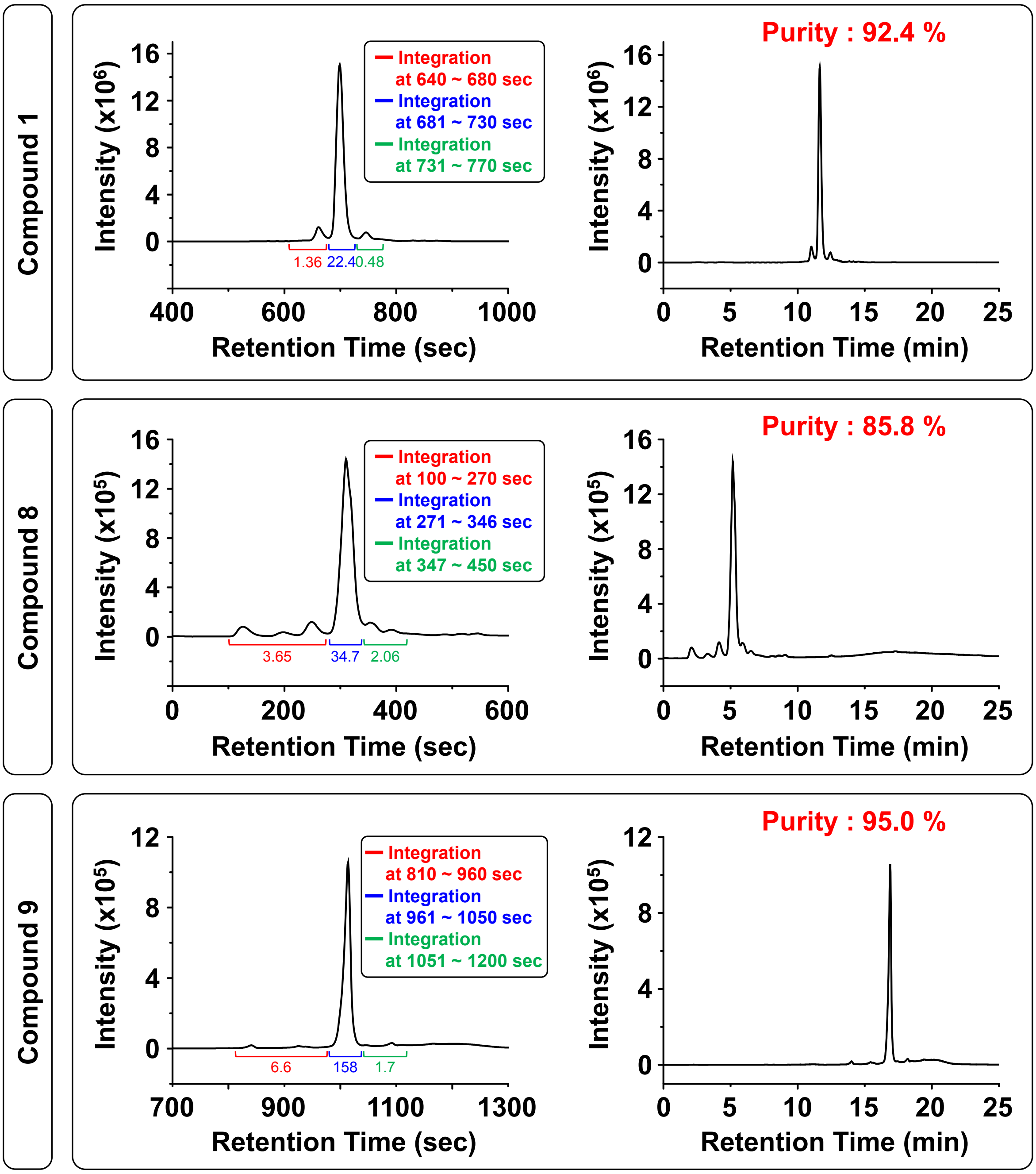

Figure S11: Purity determination of compounds **1**, **8** and **9** by HPLC (Wavelength: black 340 nm, gradient: 70 % B to 100 % B for 15 min, then 100% B during 10 min; A: Water, B: ACN).

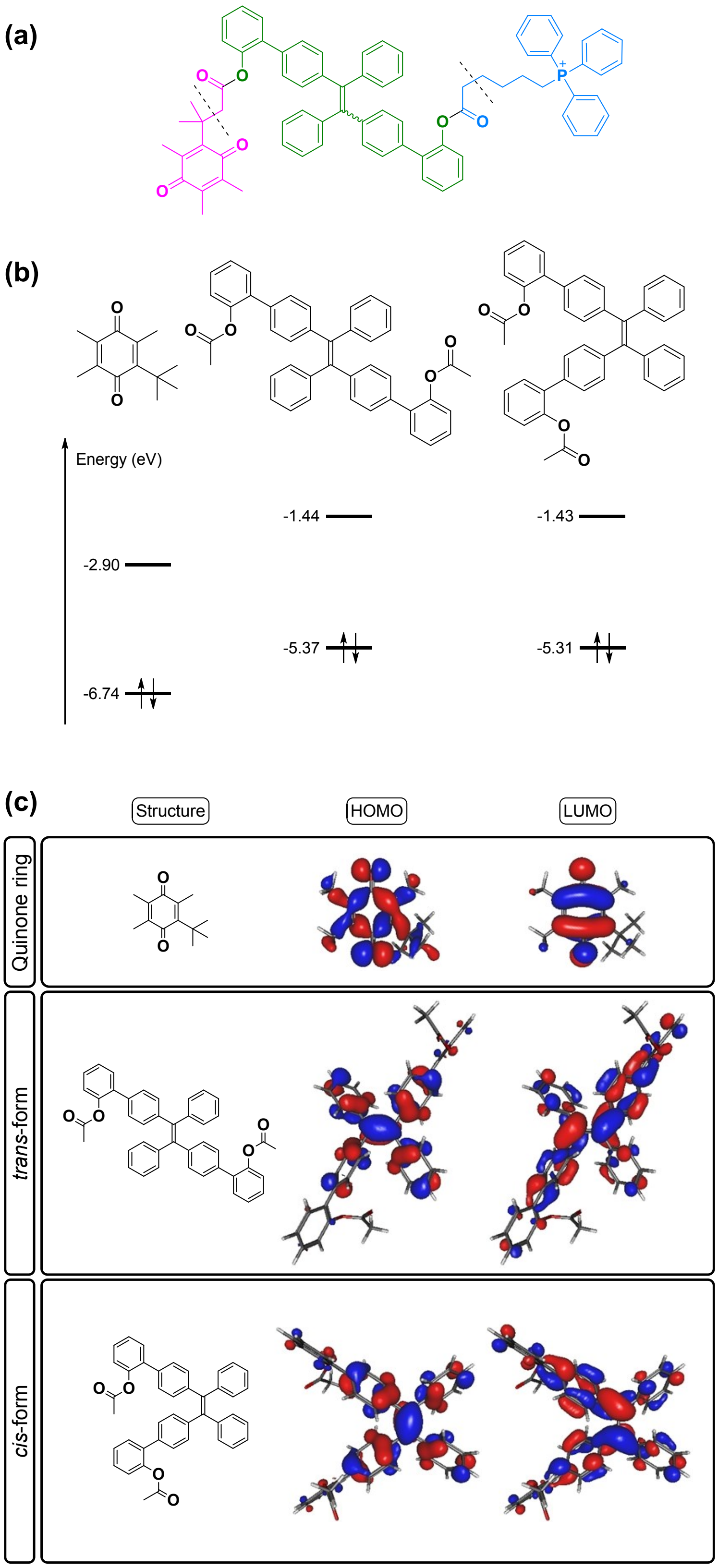

Figure S12. DFT theoretical calculations B3LYP at the 6-31G(d) level of theory. (a) The structure of **1** showing the truncation sites. (b) Energy diagram of the frontier orbitals of the trigger's quinone unit and the trans and cis substituted dyes. (c) The electron distributions of the frontier orbitals at the 0.03 e.(bohr)$^{-3}$ isovalue.

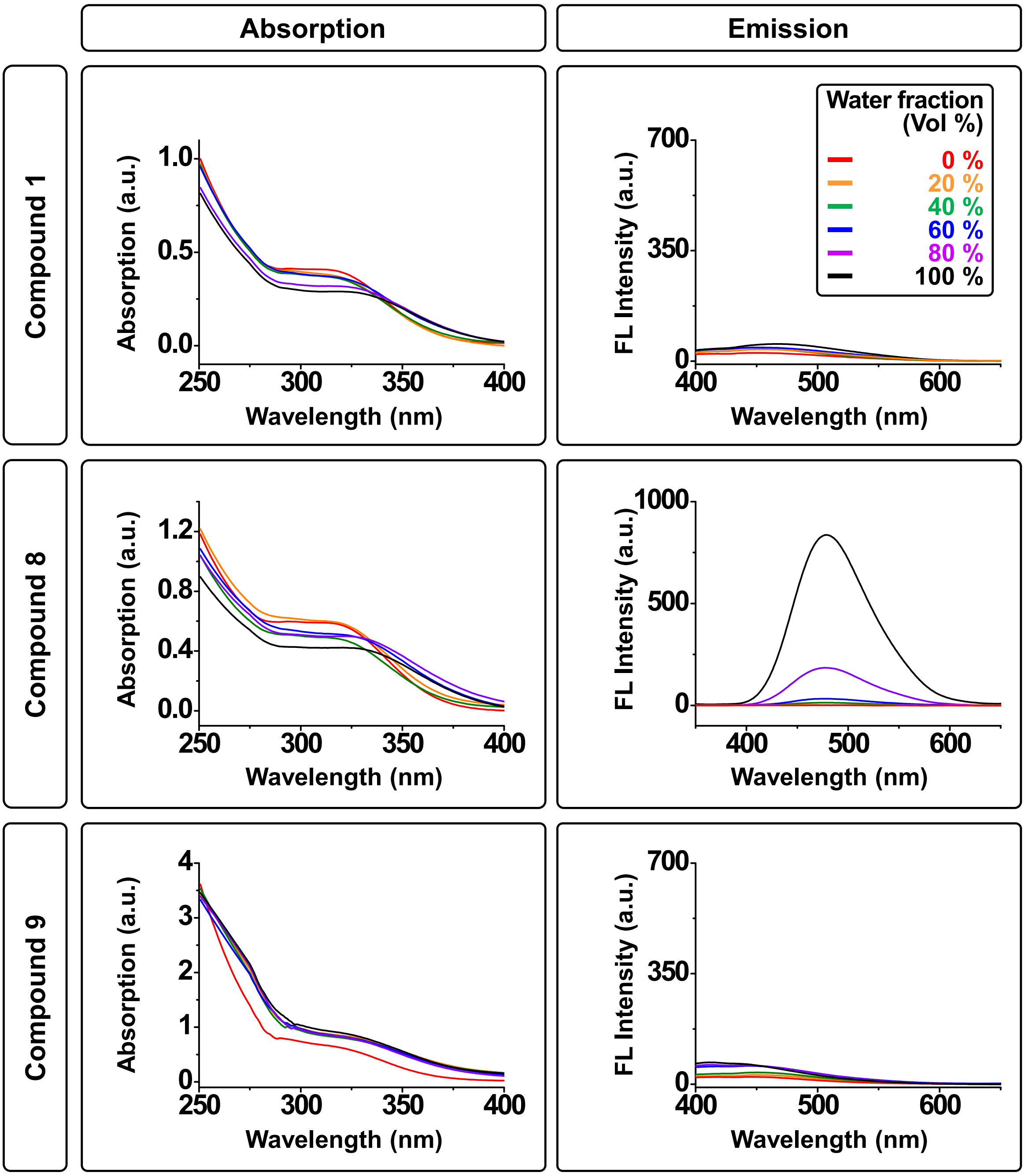

Figure S13: The dependence of water content (in methanol) on the AIE absorption of probe **1**, **8** and **9** (Concentration : 50 µM, T = 24 ˚C). emission of probe **1**, **8** and **9** (concentration : 10 µM, T = 24 ˚C, slit : 5/5 nm, sensitivity : high)

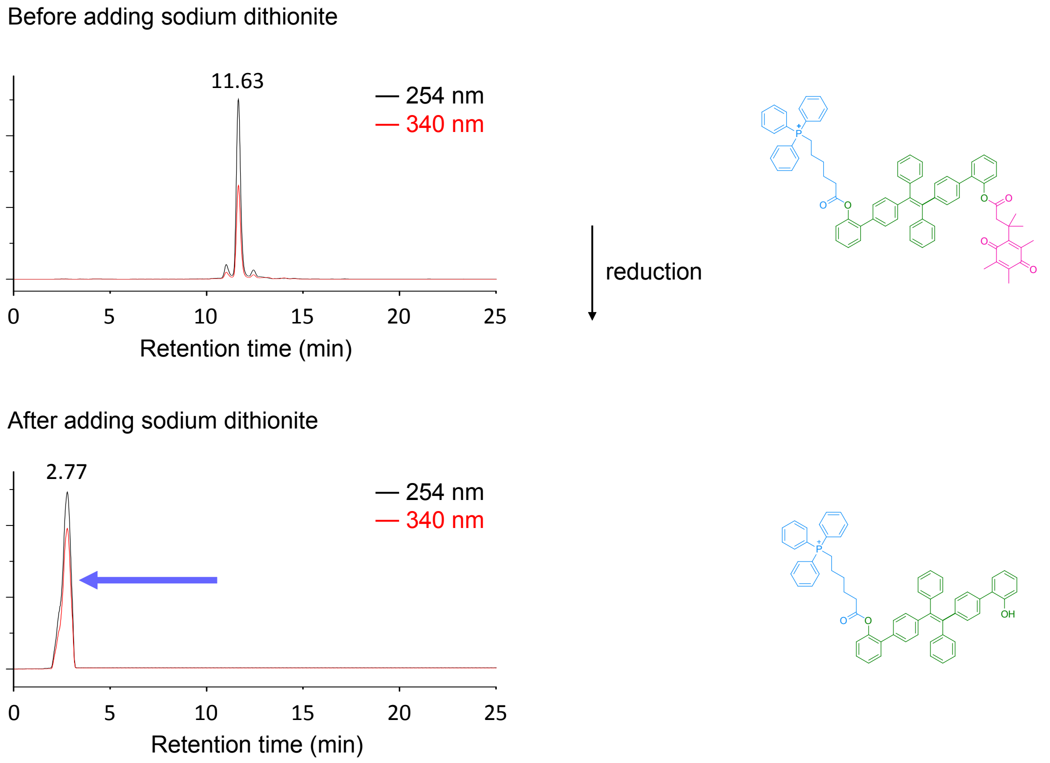

Figure S14: HPLC analysis of **1** upon the addition of sodium dithionite. (dual wavelength: black 254 nm, red 340 nm, gradient: 70 % B to 100 % B for 15 min, then 100% B during 10 min; A: Water, B: ACN).
